# Quantifying fat zonation in liver lobules: an integrated multiscale in silico model combining disturbed microperfusion and fat metabolism via a continuum biomechanical bi-scale, tri-phasic approach

**DOI:** 10.1007/s10237-023-01797-0

**Published:** 2024-02-25

**Authors:** Lena Lambers, Navina Waschinsky, Jana Schleicher, Matthias König, Hans-Michael Tautenhahn, Mohamed Albadry, Uta Dahmen, Tim Ricken

**Affiliations:** 1 Institute of Structural Mechanics and Dynamics, Faculty of Aerospace Engineering and Geodesy, University of Stuttgart, Pfaffenwaldring 27, Stuttgart, 70191 Germany; 2 Experimental Transplantation Surgery, Department of General, Visceral and Vascular Surgery, Jena University Hospital, Drackendorfer Straße 1, Jena, 07747 Germany; 3 Friedrich-Schiller-Universität Jena, Fürstengraben 27, Jena, 07743 Germany; 4 Systems Medicine of Liver, Institute for Theoretical Biology, Institute for Biology, Humboldt-University Berlin, Philippstraße 13, 10115 Berlin, Germany; 5 Department of Visceral, Transplantation, Thoracic and Vascular Surgery, University Hospital Leipzig, Liebigstraße 20, Leipzig, 04103 Germany; 6 Department of Pathology, Faculty of Veterinary Medicine, Menoufia University, Shebin Elkom, Menoufia, Egypt

**Keywords:** Multiscale approach, Homogenization, Theory of Porous Media (TPM), Growth kinematics, Metabolic dysfunction-associated steatotic liver disease (MASLD), FEM boundary problem

## Abstract

Metabolic zonation refers to the spatial separation of metabolic functions along the sinusoidal axes of the liver. This phenomenon forms the foundation for adjusting hepatic metabolism to physiological requirements in health and disease (e.g., metabolic dysfunction-associated steatotic liver disease/MASLD). Zonated metabolic functions are influenced by zonal morphological abnormalities in the liver, such as periportal fibrosis and pericentral steatosis. We aim to analyze the interplay between microperfusion, oxygen gradient, fat metabolism and resulting zonated fat accumulation in a liver lobule. Therefore we developed a continuum biomechanical, tri-phasic, bi-scale, and multicomponent in silico model, which allows to numerically simulate coupled perfusion-function-growth interactions two-dimensionally in liver lobules. The developed homogenized model has the following specifications: (i) thermodynamically consistent, (ii) tri-phase model (tissue, fat, blood), (iii) penta-substances (glycogen, glucose, lactate, FFA, and oxygen), and (iv) bi-scale approach (lobule, cell). Our presented in silico model accounts for the mutual coupling between spatial and time-dependent liver perfusion, metabolic pathways and fat accumulation. The model thus allows the prediction of fat development in the liver lobule, depending on perfusion, oxygen and plasma concentration of free fatty acids (FFA), oxidative processes, the synthesis and the secretion of triglycerides (TGs). The use of a bi-scale approach allows in addition to focus on scale bridging processes. Thus, we will investigate how changes at the cellular scale affect perfusion at the lobular scale and vice versa. This allows to predict the zonation of fat distribution (periportal or pericentral) depending on initial conditions, as well as external and internal boundary value conditions.

## Introduction

Metabolic dysfunction-associated steatotic liver disease (MASLD) poses a significant global health concern, affecting approximately 25% of individuals in Western countries (Younossi et al. 2016) with a forecast sharp increase. The Western lifestyle is characterized by reduced physical activity and an elevated intake of saturated fats and alcohol so-called western pattern diet, leading to obesity and the accumulation of fat in the liver (Friedman et al. 2018). This accumulation of fat in liver cells, specifically hepatocytes, may interfere with the physiological perfusion of the liver due to the compression of liver capillaries, the so-called sinusoids (Da Pereira et al. 2017). However, our understanding of the intricate interplay between fat accumulation and distribution in the liver, the resulting perturbation of hepatic perfusion at macro- and microscale, and the subsequent impairment of metabolic function remains limited. This knowledge gap is a barrier to progress in liver research, as it impedes the development of new diagnostic tools for assessing MASLD severity and delays the development of new prophylactic and therapeutic strategies for MASLD. Therefore, understanding the complexity of fatty liver disease, hepatic perfusion, and oxygen supply, and how this affects the metabolic function of the liver, is of paramount importance. This knowledge can assist in further developing computational tools to predict the progression of fatty liver disease, helping physicians and patients in shared decision process. On the other hand these insights and computational tools can be integrated into clinical decision support systems urgently needed in surgical oncology.

**Fig. 1:**
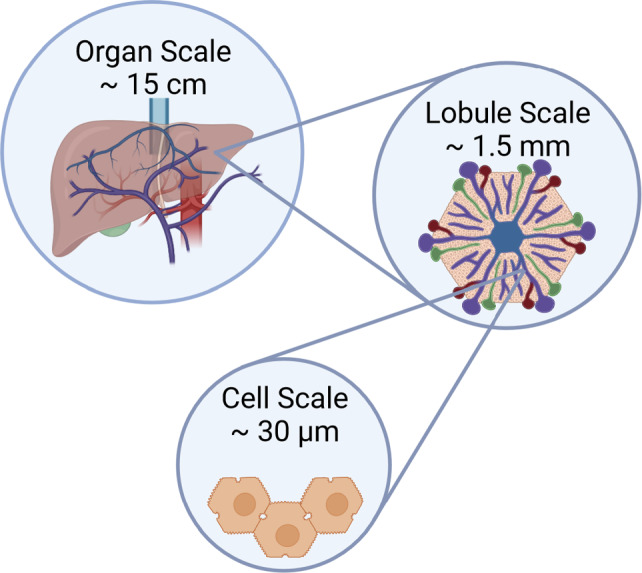
Architecture of the human liver: vascular perfusion network on the organ scale, the tissue scale of the lobule and the liver cells (hepatocytes)

In order to be able to model the liver’s complex structure on the different spatial scales, a scale-depending description of the coupled hepatic perfusion functions is required. Metabolic functions comprise, e.g., protein, carbohydrate and lipid metabolism. The metabolic processes in the liver are distributed zonally and spatially along the entire porto-central axis of the liver (Ben-Moshe et al. 2019; Gebhardt and Matz-Soja 2014). To effectively model the intricate hepatic structure, along with its corresponding functions on various spatial scales, a scale-depending description of the coupled hepatic perfusion and function is required. On the scale of the lobule, i.e., the hexagonal structured functional unit of the liver, see Fig. 1, the spatial organization of hepatic microperfusion is described. The liver and each lobule within the liver possess a dual blood supply. It is perfused with nutrient rich yet oxygen depleted blood from the portal vein, as well as oxygen rich blood from the hepatic artery. This perfusion enables transport of nutrients and oxygen to the hepatocytes. The hepatocytes are the smallest functional units in the liver, where the key metabolic processes take place (Kietzmann 2017; Ben-Moshe et al. 2019). The hepatocytes are organized along the sinusoids, forming the liver lobule. The liver lobule is characterized by three zones, the periportal zone, the mid zone and the pericentral zone. Each of these zones harbors unique metabolic functions, a phenomenon called metabolic zonation. For example, oxygen consuming processes such as fatty acid oxidation or gluconeogenesis take place predominantly in the periportal zone; whereas, glycolysis and drug metabolism are located mostly in the pericentral zone (Ben-Moshe et al. 2019; Colnot and Perret 2011). Fat accumulation in hepatocytes, which leads to a partial obstruction of the hepatic sinusoids, is causing microperfusion impairment (Da Pereira et al. 2017). It is well known that changes in perfusion, such as hyperperfusion after liver resection or hypoperfusion in the case of portal vein embolism, will affect the metabolic capacity (Kovács et al. 2018; Takemura et al. 2006; Yao et al. 2011). It has also been shown, that hepatic steatosis itself, independent of the underlying perfusion disorder, may impact zonated metabolic function (Ashworth et al. 2016). Indeed, a recent publication demonstrated that the periportal accumulation of fat in hepatocytes did influence zonal metabolic functions, such as drug metabolism (Albadry et al. 2022). Mathematical models offer valuable insights into the mechanism of hepatic perfusion, enabling improvements in diagnostic tools for physicians. By simulating the effects of liver diseases and disruptions in liver function, these models aid in understanding and diagnosing such conditions. A comprehensive overview of current computational liver models at different scales can be found, e.g., in Christ et al. (2017, 2021), Ricken and Lambers (2019). These simulations should provide results within a short time period, but should also be reliable and patient specific. Over the past years, diverse simulations and mathematical models have been published, employing various approaches including fluid dynamics, poroelastic mechanical modeling, electrical modeling, as well as scale-dependent methodologies.

To describe the processes on the lobular scale, a homogenized continuum mechanical multiphase approach using the Theory of Porous Media (TPM) has been used to characterize the interaction process between lobule and cell. This approach is based on a poroelastic multiscale model according to Ricken et al. (2010) and is enhanced by an improved transverse isotropic permeability approach to simulate hepatic blood flow. Instead of the analytical scale-bridging first order homogenization proposed here, numerical homogenization methods for porous media have also been developed recently, cf. Sandström et al. (2013), Jänicke et al. (2015), Ricken et al. (2022). However, this approach significantly increases the numerical effort, thus model order reduction methods become meaningful, cf. Armiti-Juber and Ricken (2022).

Alternatively, some mathematical models apply the Computational Fluid Dynamics (CFD) method to focus on the perfusion on the lobule scale. For example, Peeters et al. (2015) simulate the changes of macro-perfusion in a cirrhotic liver; whereas, Debbaut et al. (2014) present a CFD approach for focusing on the microperfusion in the porous liver lobule, using a multidimensional approach to estimate the anisotropic permeability. Boissier et al. (2020) developed a modeling framework to study blood flow using convection–reaction equation for transport in blood. Another approach for the simulation of hepatic processes is a compartment model using different compartments for different hepatic lobule sections. Ashworth et al. (2016) presented an 8-compartment model to simulate fat accumulation in liver lobules regarding the zonation with a higher fat development in compartments near the central vein. Given the varied size scales of the liver, Sluka et al. (2016) developed models for these different scales and integrated them into a multiscale model. This model was subsequently expanded upon by Fu et al. (2018), who compared box, pipe and net models to simulate the detoxification of xenobiotics. In addition, Wang et al. (2021) introduced a poro-viscoelastic model, focusing on tumor development within the liver lobules.

A further aspect is the simulation of transport and metabolism processes on the cell scale. The underlying processes of the development of lipid droplets have been investigated by Wallstab et al. (2017) using a kinetic model. Schliess et al. (2014) adopt a spatial-temporal model to simulate detoxification in a pre-injured liver; whereas, the approach using a multiphase multiscale enhanced TPM (eTPM) model, see Ricken et al. (2015) and Lambers (2023), focuses on the transport mechanisms and interactions between the hepatic blood and the hepatocytes. While simulating liver diseases to demonstrate the growth capability of the liver during fat accumulation leading to MASLD, several models deal with the tissue remodeling and alterations in the elasticity of the liver lobule. Waschinsky et al. (2016), Lambers et al. (2021), Lambers et al. (2019) or Lambers (2023) focus on the effects of growth due to a high-fat diet, as well as other models using a one-phasic, cf. Menzel and Kuhl (2012), or a bi-phasic, cf. Ricken and Bluhm (2009, 2010b), approach. An approach for the study of growth in liver after partial hepatectomy has been presented by Hoehme et al. (2023).

While in this work the focus lies on the cellular and hepatic lobule scale, other studies focus on the organ scale and here essentially on the description of the supplying and draining vascular systems separated across the hepatic lobules, most of them in silico, cf. Debbaut et al. (2011), Schwen et al. (2015), Rohan et al. (2018), Barléon et al. (2018), Jessen et al. (2022) and a few also experimentally, cf. Aramburu et al. (2017). An overview of computational models for blood transport in the liver across scales is provided in Ho et al. (2023).

### Structure of the work

**Fig. 2:**
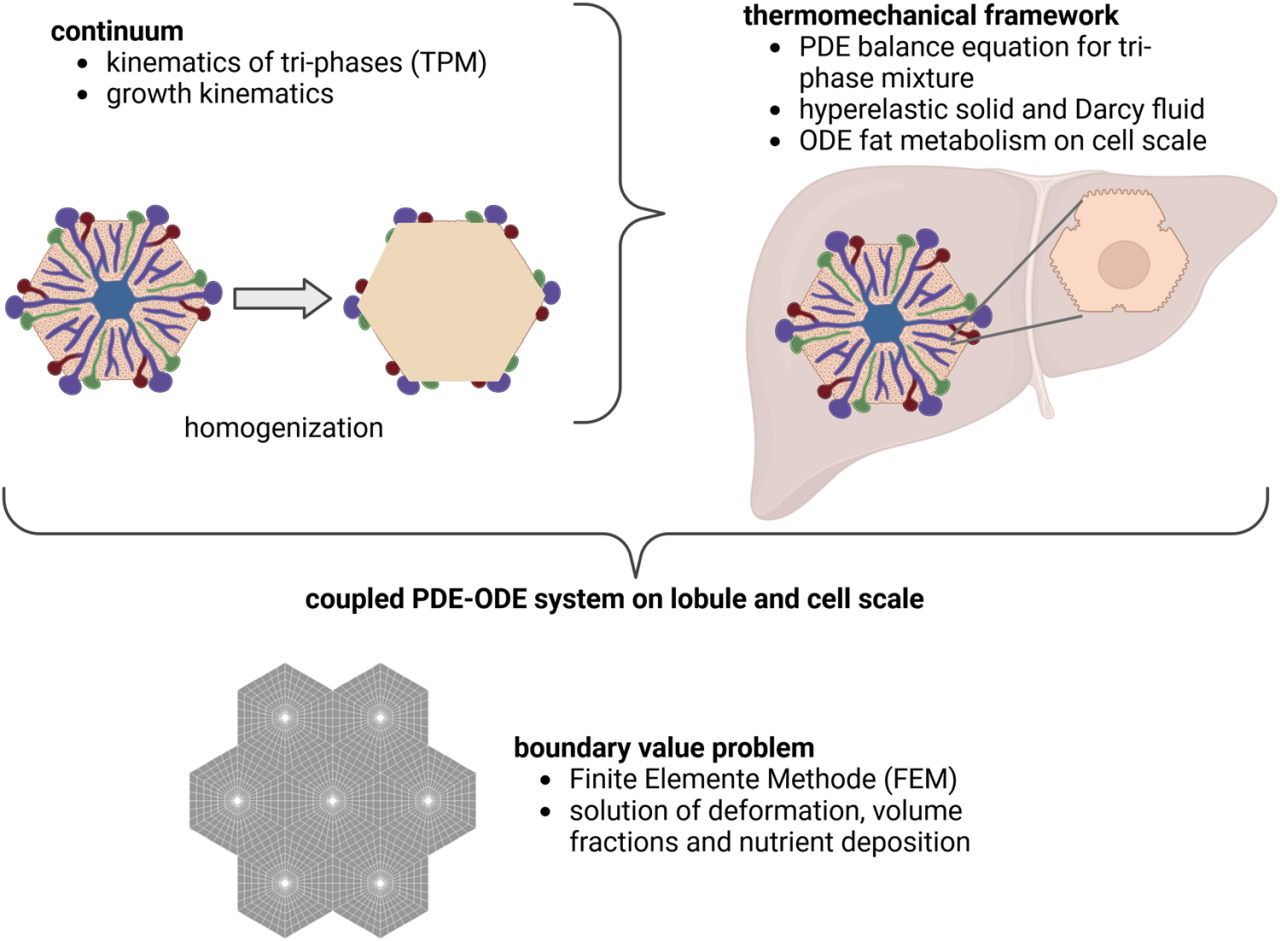
Numerical outline of the model: The continuum is described via a mixture approach with three phases, including lipid-induced tissue growth aspects. A thermodynamic evaluation gives restrictions for the material description of the solid, the perfusion of the fluid (Darcy) and the external miscible components (Fick’s Law). Furthermore, we get restrictions for the development of the cell scale. The mathematical model ends in a coupled PDE-ODE approach, which is embedded in the Finite Element Method (FEM) for the evaluation of a boundary value solution

The primary objective of this paper is to analyze the hepatic fatty acid metabolism within the porous liver structure, and to determine the impact of fat accumulation on microperfusion. Given that microperfusion and metabolic processes operate at different scales, it is reasonable to initially separate these scales when generating models. The scales are initially separated to stream line the model-building process (using a bi-scale macro-/micro-approach), and then coupled again via embedding during the numerical calculations. To model the coupled fluid-tissue interaction behavior in the lobule, we apply the Theory of Porous Media (TPM), a continuum biomechanical based homogenization mixture approach, which allows the consideration of multiple components. Hence, the characterization of the fluid in the micro-vessels (so-called sinusoids) is realized via a homogenized transvers-isotropic flow permeability description, cf. Pierce et al. (2013a), Wang et al. (2018), enhanced with diffusion and advection components, cf. Ricken et al. (2020). Considering the resulting restrictions from the enlarged entropy inequality evaluation, we define thermodynamically consistent constitutive relations for the blood-saturated poroelastic material behavior, cf. Ricken et al. (2015). The cell scale describes the time-dependent change in metabolites due to biochemical reactions (hepatic metabolism). For this purpose, we created a system of ordinary differential equations (ODE) corresponding to the rate of changes due to hepatic processes. For thermodynamic consistency, we formulate the constitutive restrictions for the cell metabolism regarding the mass-exchange in the liver cell, cf. Ricken et al. (2015). Focusing on the key metabolic pathways involved in fat accumulation in the liver, we have included the glucose and fatty acid metabolism. Both scales are weakly coupled (micro to macro) by computing the dynamics of the nutrients $\hat{\rho }^{\alpha \beta }$ in each Gauss point and time step, see Chapter 3. Furthermore, the coupled equation system is solved using a spatial-temporal resolved PDE-ODE-model in the Finite Element Method (FEM), computing the stress-deformation behavior and including the possibility of lipid growth, cf. Ateshian and Ricken (2010), Ricken and Bluhm (2009), Ricken et al. (2007). The hepatic fat metabolism, depending on perfusion, oxygen saturation and metabolite gradients, is investigated and analyzed by our modeling approach. This provides insights into the mechanisms responsible for an inhomogeneous (zonated) accumulation of fat during fatty liver degeneration. Figure 2 illustrates the numerical approach used for the studies. In the first part, the macro-level is evaluated. First, the mixture approach with the resulting kinematics is discussed. Then, the thermodynamic framework is evaluated and the consequences for the restriction of the stresses, interaction forces and mass-exchange are developed. The second part describes the fat metabolism on the microscale. In a FE-implementation of this approach, we provide a showcase describing the coupling of perfusion disturbance and tissue growth. This growth occurs during the development of a metabolic dysfunction-associated steatotic liver disease (MASLD), a widely spread disease caused, among others, by an excessive uptake of free fatty acids (FFAs) resulting in the accumulation of triglycerides (TGs) in the liver cells (Friedman et al. 2018). This growth decreases the blood perfusion via the formation of lipid droplets and thus can impair liver metabolism and functionality due to alterations in supply or drainage (Farrell et al. 2008). Computing this scenario allows a better understanding of the fatty liver pathology and its effect on liver functionality.

## Macro-model: derivation of a tri-phase approach for the lobule scale

The human liver presents a complex architecture. On the organ level, the liver contains a complex vascular blood supply and drainage system which changes on the different scales from a vascular branching tree to micro-vessels. Our basic liver model focuses on the processes in the liver lobule. The lobule is composed of a portal triad (artery, portal vein and bile duct) containing the blood inflow, the central vein as the outflow of the anisotropically arranged sinusoids and cells, mainly the hepatocytes positioned along the micro-vessels. Blood from the portal triad enters the sinusoids on the lobule scale. From a mechanical point of view, we have to deal with coupled fluid-solid interactions including microscopic components, which are transported via the fluid. For the formulation of the mixture approach, we apply the TPM, which is based on the works of, e.g., Boer (2000), De Boer (1996) and Ehlers (2002). This approach has been widely used for the description of porous biological materials, cf. Ehlers and Markert (2001). Mass transfer processes have been added to TPM to describe phase transitions between constituents, cf., e.g., Ehlers and Häberle (2016) or Ricken and Bluhm (2010a) for non-miscible phases and, e.g., Robeck et al. (2011), Ricken et al. (2014) or Ricken and Thom (2020) for miscible components.

This approach includes the description of three main phases (solid, fat and fluid) which are immiscible and heterogeneously composed. The fluid description is set as an anisotropic perfusion model and behaves as a Darcy flow depending on the permeability and the applied pressure, see Ricken et al. (2010). Moreover, the mixture includes miscible substances resulting in a multicomponent model, see Ricken et al. (2014) or Seyedpour et al. (2019, 2021b). The miscible substances play an important role in the metabolism processes and are characterized analogous to Fick’s law. We distinguish external nutrients (included in the fluid phase) and internal substances (included in the solid phase).

### The Theory of Porous Media

The biological tissue of the human liver is a soft porous tissue of a complex architecture consisting of different components perfused by blood. Thus, a heterogeneous and discontinuous structure exists. The eTPM is an applicable approach to average the geometry into a continuous structure, which leads to a mixture formulation considering the concept for volume fraction. With this, we are able to compute the motion and interaction of each individual phase including phase transitions. Moreover, the proposed description is based on the fundamental axioms of mechanics and thermodynamics. In addition, the approach provides a reasonable framework for growth aspects, see, e.g., Ambrosi et al. (2016), Steeb and Diebels (2003), Amar and Goriely (2005) or Humphrey (2002). In terms of the computation of the macroscale, we apply the TPM for a mathematical description of the liver lobule as a biological structure, including the porous liver tissue with heterogenic fat distribution and blood supply.

To reduce the numerical effort, we assume some simplifications. Since the compressibility of the liver, fat-tissue, and blood is much smaller than that of the entire porous body, we can interpret the applied phases as materially incompressible, with a constant density for the liver tissue and fat-tissue $(\rho ^{\textrm{SR}})^{\prime }_{\textrm{S}}\,=\,\textrm{0},\, (\rho ^{\textrm{TR}})^{\prime }_{\textrm{S}}\,=\,\textrm{0}$ as well as $(\rho ^{\textrm{FR}})^{\prime }_{\textrm{S}}\,=\,\textrm{0}$, where the real density of the phase $\alpha$ is denoted by $\rho ^{\alpha \mathrm R}$. Furthermore, we assume only isothermal processes with the same temperature for the phases $\theta ^{\textrm{S}}\,=\,\theta ^{\textrm{T}}\,=\,\theta ^{\textrm{F}}= \rm const.$ with $(\theta ^{\alpha })^{\prime }_{\alpha }\,=\,\textrm{0}$, so that no energy exchanges between the phases can occur, with ${\hat{\text e}}^{\mathbf{\alpha }}=0$. Finally, a transient but quasi static description of motion is chosen, where the acceleration $\textbf{x}^{\prime \prime }_{\alpha }\,=\,\textbf{0}$.

**Fig. 3:**
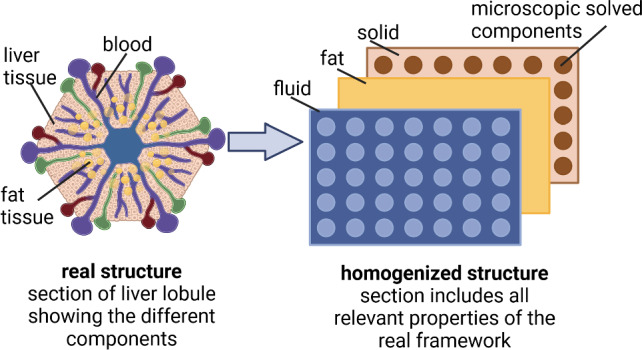
Mixture approach: outline of the homogenization of the biological components

The objective of the mixture theory is to design a substitute model for the formulation of the real structure on the macroscale, including microscopic properties. The true structure includes many different components, for example different aggregate phases or concentrations for which we assume an ideal disorder over the control space. The physical and chemical properties of the microstructure are replaced by averaged and "smeared" properties on the macroscale. Motion, deformation and stresses are computed as statistically averaged values of the real structure in the control space. Such a mixture describes a heterogeneous continuum containing the different components and their interaction (Fig. 3).

The continuum is treated as an immiscible mixture of three main phases $\varphi ^{\varvec{\alpha }}$ with their particles $\textrm{X}_{\varvec{\alpha }}$ and miscible solutes $\varphi ^{\alpha \beta }$ with their particles $\textrm{X}_{\alpha \beta }$, which exist in each solvent $\varphi ^{\alpha }$. The overall structure is the mixture of all included components, so the whole system $\varphi$ can be described as

1 $$\begin{aligned} \varphi = \displaystyle \sum \limits _{\alpha =1}^\kappa \varphi ^{\varvec{\alpha }}:= \displaystyle \sum \limits _{\alpha =1}^\kappa (\displaystyle \sum \limits _{\beta =1}^{\nu -1} (\varphi ^{\alpha \beta }) + \varphi ^{\alpha } ). \end{aligned}$$

Where $\kappa \,=\,\textrm{3}$ shows the main immiscible carrier phases $\varphi ^{\varvec{\alpha }}$ with

2 $$\begin{aligned} \varphi ^{\varvec{\alpha }}=\{\mathrm {S,T,F\} } =\varvec{\alpha }_{\textrm{i}}\,\mathrm {\vert i=1..3} \end{aligned}$$

and $\mathrm {(\nu -1)}$ the microscopic components, which are solved in the carrier phases. The hyper-elastic tissue solvent of the liver includes the microscopic solutes glycogen $\varphi ^{\mathrm{S\beta }}$ and the blood solvent contains the solutes glucose, lactate, free fatty acids (FFA) and oxygen $\varphi ^{\mathrm{F\beta }}$.

3 $$\begin{aligned} \begin{array}{lclcl} \varphi ^{\mathrm{S\beta }}\,&{}=&{}\,{\textrm{Gy}}\,&{}=&{}\,{\mathrm{\beta _i}}\,{\mathrm{\vert i=1}} \\ \varphi ^{\mathrm{F\beta }}\, &{}=&{}\,{\textrm{Gu,Lc,FFA,Ox}}\,&{}=&{}\,{\mathrm{\beta _i}}\,{\mathrm{\vert i=1..4}} \end{array} \end{aligned}$$

For evaluating the volume of the different phases, the mixture theory is enhanced by the concept of volume fractions yielding the saturation condition. The overall volume $\textrm{V}$ of the whole mixture can be calculated via the sum of the partial volume $\textrm{dv}^{{\alpha }}$ as

4 $$\begin{aligned} \begin{aligned} \textrm{V}&= \displaystyle \sum \limits _{\varvec{\alpha }=\varvec{1}}^{\varvec{\kappa }}\,\textrm{dv}^{\varvec{\alpha }}= \displaystyle \int \limits _{\textrm{Bs}} \displaystyle \sum \limits _{\varvec{\alpha }=\varvec{1}}^{\varvec{\kappa }}\,\textrm{dv}^{\varvec{\alpha }}= \displaystyle \int \limits _{\textrm{Bs}} \displaystyle \sum \limits _{\varvec{\alpha }=\varvec{1}}^{\varvec{\kappa }}\,\textrm{n}^{\varvec{\alpha }}\,\textrm{dv}\\&\text {with} \quad \varvec{\kappa }\, \in \, \{\mathrm {S,T,F\}}. \end{aligned} \end{aligned}$$

Equation (4) leads to the saturation condition, which is composed of the volume fraction $\textrm{n}^{\varvec{\alpha }}$, obtained as

5 $$\begin{aligned} \displaystyle \sum \limits _{\varvec{\alpha }=\varvec{1}}^{\varvec{\kappa }} \textrm{n}^{\varvec{\alpha }}= 1 \,\, \text {with} \quad \varvec{\kappa }\, \in \, \{\mathrm {S,T,F\}}. \end{aligned}$$

For an effective connection between the balance equations, the volume fractions and the saturation condition, we introduce the density of the phases. Here, we can distinguish between the partial density $\rho ^{\varvec{\alpha }}$, resulting from the overall volume $\textrm{V}$ and the realistic density $\rho ^{\varvec{\alpha }\textrm{R}}$, applying the partial volume $\textrm{dv}^{\varvec{\alpha }}$ of the phases by using the partial mass $\rm dm^{\varvec{\alpha }}$:

6 $$\begin{aligned} \begin{array}{lcl} \mathrm {\rho ^{\varvec{\alpha}}}=\mathrm {\displaystyle \frac{dm^{\varvec{\alpha }}}{dv}},\hspace{0.5cm} \mathrm {\rho ^{\varvec{\alpha }R}}=\mathrm {\displaystyle \frac{dm^{\varvec{\alpha }}}{dv^{\varvec{\alpha }}}} . \end{array} \end{aligned}$$

Furthermore, we can connect the partial density to the volume fractions of the phases obtained as

7 $$\begin{aligned} \mathrm {\rho ^{\varvec{\alpha }}=\displaystyle \frac{dm^{\varvec{\alpha }}}{dv} =\displaystyle \frac{dm^{\varvec{\alpha }}}{dv^{\varvec{\alpha }} / n^{\varvec{\alpha }}} =n^{\varvec{\alpha }} \ \rho ^{\varvec{\alpha }R} }. \end{aligned}$$

The microscopic components included in the phases are described with their concentration $\textrm{c}^{\alpha \beta }$ and molecular weight $\textrm{M}^{\beta }_\textrm{mol}$. The concentration is obtained by the fraction of the number of moles $\rm {dn}^{\beta }_\text{mol}$ to the partial volume and the molecular weight is developed by the fraction of the mass of the component $\textrm{dm}^{\beta }$ to the number of moles:

8 $$\begin{aligned} \begin{array}{lcl} \textrm{c}^{\alpha \beta }=\displaystyle \frac{\textrm{dn}^{\beta }_{\rm mol}}{\textrm{dv}^{{\alpha }}}, \hspace{0.5cm}\textrm{M}^{\beta }_{\rm mol}=\displaystyle \frac{\textrm{dm}^{\beta }}{\textrm{dn}^{\beta }_{\rm mol}}. \end{array} \end{aligned}$$

The partial molar density $\rho ^{\alpha \beta }$ of the microscopic components results from the multiplication of the volume fraction with the concentration and the molecular weight of the constituent. Hence, we can introduce the true molar density $\rho ^{\alpha \beta \mathrm R}$ with:

9 $$\begin{aligned} \rho ^{\alpha \beta }\,=\,\textrm{n}^{\alpha }\,\textrm{c}^{\alpha \beta }\,\textrm{M}^{\beta }_\textrm{mol}\,=\,\textrm{n}^{\alpha }\,\rho ^{\alpha \beta \mathrm R}. \end{aligned}$$

### Kinematics of the multiphase approach

**Fig. 4:**
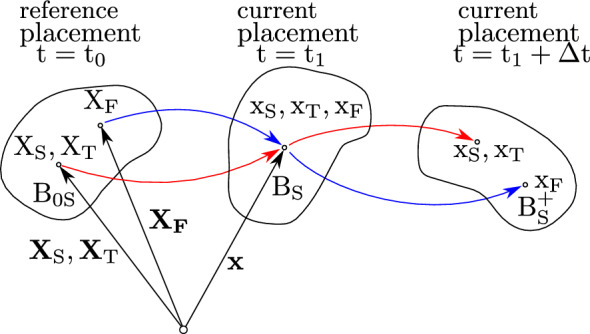
Outline of the particle motion for the solid tissue $\rm X_\text S$, fat-tissue $\rm X_{\text T}$ and blood $\rm X_\text F.$

Figure 4 shows the outline of the movement of the particles in the control space. Each material point is simultaneously occupied by all components. Assuming that the particles $\textrm{X}_{\varvec{\alpha }}$ simultaneously occupy the current placement of the solid at time $\textrm{t} = \textrm{t}_{\textrm{1}}$ in the body $\rm B_\text S$ results in different particle reference positions at time $\textrm{t} = \textrm{t}_{\textrm{0}}$ in the reference body $\textrm{B}_{\textrm{0S}}$, since they assign their own independent motion function. In detail, we assume an independent motion function for the carrier phases as well as the solutes. Moreover, it is assumed that the solid $\textrm{X}_{\textrm{S}}$ and fat $\textrm{X}_{\textrm{T}}$ particles have the same motion function.

Thus, the independent motion functions of the main phases ${\varvec{\chi }}_{\small {\varvec{\alpha }}}$ follow as

10 $$\begin{aligned} {\textbf {x}}={\varvec{\chi }}_{\small {\varvec{\alpha }}}(\textbf{X}_{\varvec{\alpha }},\textrm{t})\, \hspace{0.8cm}\textbf{x}\,\in \,\textrm{B}_{\textrm{S}}. \end{aligned}$$

The velocity of the three phases ${\textbf{x}}'_{\hspace{-0.02cm}\varvec{\alpha }}$ is defined as the material time derivation of the motion obtained as

11 $$\begin{aligned} {\textbf{x}}'_{\hspace{-0.02cm}\varvec{\alpha }}=\frac{\partial {\varvec{\chi }}_{\small {\varvec{\alpha }}}(\textbf{X}_{\varvec{\alpha }},\textrm{t})}{\partial \textrm{t}}. \end{aligned}$$

### Growth Kinematics

The eTPM enables the description of tissue growth processes, which result from a mass-exchange between components. Additionally, for the fat phase we introduce the multiplicative split of the deformation gradient $\textbf{F}_{\textrm{T}}$ into a growth part $\textbf{F}_{\textrm{T}}^\textrm{g}$ and an elastic part $\textbf{F}_{\textrm{T}}^\textrm{e}$ with $\textbf{F}_{\textrm{T}}= \textbf{F}_{\textrm{T}}^\textrm{e}\, \textbf{F}_{\textrm{T}}^\textrm{g}$ according to Rodriguez et al. (1994). To consider the interaction between the phases in the mixture, the balance of mass contains the mass-exchange term $\hat{\rho }^{\alpha }$, which is equal to the rate of the mass. In this context, the growth is restricted to the fat-tissue phase with the density $\rho ^{\textrm{T}}$ resulting in the mass balance

12 $$\begin{aligned} (\rho ^{\textrm{T}})^{\prime }_{\textrm{T}}\,+\rho ^{\textrm{T}}\,\frac{(\textrm{J}_{\textrm{T}})'_{\textrm{T}}}{\textrm{J}^{\textrm{T}}}\,=\,\hat{\rho }^{\textrm{T}}, \end{aligned}$$

also see Appendix 7.1. The underlying processes of the fat metabolism are explained in Chapter 3.

### Field equation

The eTPM approach of the macroscopic liver lobule has to fulfill the physical requirements of the balance equations of mass, momentum and of angular momentum. The model takes into account an incompressible solid phase $\varphi ^{\textrm{S}}$ and fat phase $\varphi ^{\textrm{T}}$, which are derived by the same motion function, and an incompressible fluid phase $\varphi ^{\textrm{F}}$. The main phases contain substances $\varphi ^{\alpha \beta }$, which are included in the cell scale and allow phase transition to build up the metabolism. We assume fat growth initiated by the accumulation of nutrients. Considering a tri-phasic porous model with microscopic components requires additional mixture conditions, see Truesdell (1984). According to that, we include mass-exchange in the fat phase $\hat{\rho }^{\textrm{T}}$ as well as for the microscopic components $\hat{\rho }^{\alpha \beta }$ and interaction forces ${\hat{{\textbf {p}}}}^{\alpha}$ and ${\hat{{\textbf {p}}}}^{\alpha \beta }$. For the calculation of the presented model, we use the following independent relations. We apply the local form of the balance equation of mass for each main phase and microscopic component with:

13 $$\begin{aligned} \begin{array}{lll} \textrm{solid}\,&{}\,(\textrm{n}^{\textrm{S}})^{\prime }_{\textrm{S}}\,+\,\textrm{n}^{\textrm{S}}\,\textrm{div}\,\textbf{x}^{\prime }_{\textrm{S}}\,=\,\textrm{0}, \\ \textrm{fat}\,&{}\,(\textrm{n}^{\textrm{T}})^{\prime }_{\textrm{S}}\,+\,\textrm{n}^{\textrm{T}}\,\textrm{div}\,\textbf{x}^{\prime }_{\textrm{S}}\,=\,\displaystyle {\frac{\textrm{1}}{\rho ^{\textrm{TR}}}}\,\hat{\rho }^{\textrm{T}}, \\ \textrm{fluid}\,&{}\,(\textrm{n}^{\textrm{F}})^{\prime }_{\textrm{F}}\,+\,\textrm{n}^{\textrm{F}}\,\textrm{div}\,\textbf{x}^{\prime }_{\textrm{F}}\,=\,\textrm{0}, \\ \textrm{solid}\, \mathrm{comp.}\,&{}\,(\textrm{n}^{\textrm{S}})^{\prime }_{\textrm{S}}\,\textrm{c}^\mathrm{S\beta }\,\textrm{M}^{\textrm{S}\beta }_\textrm{mol}\,+\,\textrm{n}^{\textrm{S}}\,(\textrm{c}^{\textrm{S}\beta })^{\prime }_{\textrm{S}\beta }\,\textrm{M}^{\textrm{S}\beta }_\textrm{mol}\, \\ &{}+\,\textrm{n}^{\textrm{S}}\,\textrm{c}^\mathrm{S\beta }\,\textrm{M}^{\textrm{S}\beta }_\textrm{mol}\,\textrm{div}\,\textbf{x}^{\prime }_{\textrm{S}}\,=\,\hat{\rho }^{\textrm{S}\beta }\\ {}\textrm{fluid}\, \mathrm{comp.}\,&{}\,(\textrm{n}^{\textrm{F}})^{\prime }_{\textrm{F}}\,\textrm{c}^\mathrm{F\beta }\,\textrm{M}^{\textrm{F}\beta }_\textrm{mol}\,+\,\textrm{n}^{\textrm{F}}\,(\textrm{c}^{\textrm{F}\beta })^{\prime }_{\textrm{F}\beta }\,\textrm{M}^{\textrm{F}\beta }_\textrm{mol}\, \\ &{}+ \, \textrm{grad}\,{\textrm{n}^{\textrm{F}}}\,\textbf{w}_{\textrm{F}\beta \textrm{S}}\,\textrm{c}^\mathrm{F\beta }\,\textrm{M}^{\textrm{F}\beta }_\textrm{mol}\\ &{}-\,\textrm{grad}\,{\textrm{n}^{\textrm{F}}}\,\textbf{w}_{\textrm{FS}}\,\textrm{c}^\mathrm{F\beta }\,\textrm{M}^{\textrm{F}\beta }_\textrm{mol}\, \\ {} &{}+ \,\textrm{n}^{\textrm{F}}\,\textrm{c}^\mathrm{F\beta }\,\textrm{M}^{\textrm{F}\beta }_\textrm{mol}\,\textrm{div}\,\textbf{x}^{\prime }_{\textrm{F}\beta }\, =\,\hat{\rho }^{\textrm{F}\beta }. \end{array} \end{aligned}$$

The seepage velocities $\textbf{w}_{\textrm{F}\beta \textrm{S}}$ and $\textbf{w}_{\textrm{FS}}$ describe the relative velocity of the external concentrations $\textbf{x}^{\prime }_{\textrm{F}\beta }$ and the fluid velocity $\textbf{x}^{\prime }_{\textrm{F}}$ with respect to the solid velocity ${\hat{{\textbf { p}}}}^{\prime }_{\textrm{S}}$. Additionally, we assume no transport of solutes in the tissue phases. The balance of momentum achieves an equilibrium between the time derivation of the motion quantities and the sum of the forces. In the framework of TPM, the phase and nutrient interactions lead to additional interaction forces ${\hat{{\textbf { p}}}}^{{\varvec{\alpha }}}$ and ${\hat{{\textbf {p}}}}^{\alpha \beta }$, cf. Truesdell (1984).

14 $$\begin{aligned} \begin{array}{llcc} \textrm{solid}\,&{}\,\textrm{div}\,{\textbf {T}}^{\textrm{S}}\, + \, \rho ^{\textrm{S}}\, \textbf{b}^{\textrm{S}}\, + \,{\hat{{\textbf { p}}}}^{\textrm{S}}\, - \, \hat{\rho }^{\textrm{S}}\, \textbf{x}^{\prime }_{\textrm{S}}&{}=&{}\textbf{0}, \\ \textrm{fat}\,&{}\,\textrm{div}\,{\textbf {T}}^{\textrm{T}}\, + \, \rho ^{\textrm{T}}\, \textbf{b}^{\textrm{T}}\, + \,{\hat{{\textbf { p}}}}^{\textrm{T}}\, - \, \hat{\rho }^{\textrm{T}}\, \textbf{x}^{\prime }_{\textrm{S}}&{}=&{}\textbf{0}, \\ \textrm{fluid}\,&{}\,\textrm{div}\,{\textbf {T}}^{\textrm{F}}\, + \, \rho ^{\textrm{F}}\, \textbf{b}^{\textrm{F}}\, + \,{\hat{{\textbf {p}}}}^{\textrm{F}}\, - \, \hat{\rho }^{\textrm{F}}\, \textbf{x}^{\prime }_{\textrm{F}}&{}=&{}\textbf{0}, \\ \mathrm{comp.}\,&{}\,\textrm{div}\,{\textbf {T}}^{{\textrm{S}}\beta }\, + \, \rho ^{\textrm{S}\beta }\, \textbf{b}^{\textrm{S}}\, + \,{\hat{{\textbf { p}}}}^{\textrm{S}\beta }\, - \, \hat{\rho }^{\textrm{S}\beta }\, \textbf{x}^{\prime }_{\textrm{S}}&{}=&{}\textbf{0}, \\ \,&{}\, \textrm{div}\,{\textbf {T}}^{{\textrm{F}}\beta }\, + \, \rho ^{\textrm{F}\beta }\, \textbf{b}^{\textrm{F}}+ \,{\hat{{\textbf { p}}}}^{\textrm{F}\beta }\, - \, \hat{\rho }^{\textrm{F}\beta }\, \textbf{x}^{\prime }_{\textrm{F}}&{}=&{}\textbf{0}. \end{array} \end{aligned}$$

In (14), ’div’ denotes the spatial divergence operator of $\textbf{T}^{\alpha }$ and $\textbf{T}^{\alpha \beta }$, which are the partial Cauchy stress tensors of the main phases and solutes. Besides the balance equations, we apply the saturation condition

15 $$\begin{aligned} \textrm{n}^{\textrm{S}}\,+\,\textrm{n}^{\textrm{T}}\,+\,\textrm{n}^{\textrm{F}}\,=\,\textrm{1}, \end{aligned}$$

and the relation of the partial and the real density

16 $$\begin{aligned} \textrm{n}^{\textrm{S}}\,=\,\displaystyle \frac{\rho ^{\textrm{S}}}{\rho ^{\textrm{SR}}},\hspace{0.5cm} \textrm{n}^{\textrm{T}}\,=\, \displaystyle \frac{\rho ^{\textrm{T}}}{\rho ^{\textrm{TR}}},\hspace{0.5cm} \textrm{n}^{\textrm{F}}\,=\, \displaystyle \frac{\rho ^{\textrm{F}}}{\rho ^{\textrm{FR}}}. \end{aligned}$$

Furthermore, we apply the conditions for the mass-exchange between the components with:

17 $$\begin{aligned} \hat{\rho }^{\textrm{S}}\,+\,\hat{\rho }^{\textrm{T}}\,+\,\hat{\rho }^{\textrm{F}}\,+\,\hat{\rho }^{\alpha \beta }=\,\textrm{0}, \end{aligned}$$

and the interaction forces

18 $$\begin{aligned} {\hat{{\textbf { p}}}}^{\textrm{S}}\,+\,{\hat{{\textbf { p}}}}^{\textrm{T}}\,+\,{\hat{{\textbf {p}}}}^{\textrm{F}}\,+\,{\hat{{\textbf {p}}}}^{\alpha \beta }\,=\,\mathrm{\textbf{0}}. \end{aligned}$$

In order to close the system of equations, we introduce the following set of constitutive descriptions,

19 $$\begin{aligned} \mathcal {C} = [\mathrm{\textbf{T}}^{\textrm{S}}_{\textrm{sym}},\,\mathrm{\textbf{T}}^{\textrm{F}}_{\textrm{sym}},\,\mathrm{\textbf{T}}^{\mathrm{F\beta }}_{\textrm{sym}},\,{\hat{{\textbf {p}}}}^{\textrm{F}},\,{\hat{{\textbf {p}}}}^{\textrm{F}\beta },\,\hat{\rho }^{\alpha \beta }]. \end{aligned}$$

### Thermodynamically consistent constitutive relations

The second law of thermodynamics implies that the entropy in an isolated system never decreases. Evaluating a thermodynamically consistent material model requires consideration of the first and second thermodynamic law. A detailed description of the mathematical evaluation of the entropy inequality is given in the "Evaluation of the Entropy Inequality" Section 7.2 of Appendix. After identifying the elastic and dissipative processes in the entropy inequality in Section 7.3 in the Appendix, we receive restrictions for the stresses and interaction forces.

Here, the evaluation of the elastic part of the entropy inequality yields restrictions for the stresses of the solid and fluid phases. By applying a Helmholtz free energy function for a Neo Hookean solid, the following stress equations are derived

20 $$\begin{aligned} \begin{array}{lcll} \textbf{T}^{\textrm{S}}\,+\,\textbf{T}^{\textrm{T}}\,+\,\textbf{T}^{\textrm{S}\beta }\, &{}=&{}\,\displaystyle {\frac{\rho ^{\textrm{S}}}{\rho ^{\textrm{S}}_{\textrm{0S}}}}\left( \lambda ^{\textrm{S}}\,\textrm{ln}\,\textrm{J}_{\textrm{S}}\,\textbf{I}\,+\,\textrm{2}\,\mu ^{\textrm{S}}\,\textbf{K}_{\textrm{S}}\right) \, \\ {} &\quad\quad{}+&{}\,\displaystyle {\frac{\rho ^{\textrm{T}}}{\rho ^{\textrm{T}}_{\textrm{0S}}}}\left( \lambda ^{\textrm{T}}\,\textrm{ln}\,\textrm{J}_{\textrm{S}}\,\textbf{I}\,+\,\textrm{2}\,\mu ^{\textrm{T}}\,\textbf{K}_{\textrm{S}}\right) \, \\ &{}-&{}\,\lambda \,\textbf{I}\,(\,\textrm{n}^{\textrm{S}}\,+\,\textrm{n}^{\textrm{T}}) \\ \textbf{T}^{\textrm{F}}&\quad{}=\quad&{}\,-\textrm{n}^{\textrm{F}}\,(\lambda \,+\mathrm{\Pi })\,\textbf{I}\\ \textbf{T}^{\textrm{F}\beta }&\quad{}=&{}\,\textrm{n}^{\textrm{F}}\,\textrm{c}^\mathrm{F\beta }\textrm{R}\,\theta \,\textbf{I}\,=\quad\,\textrm{n}^{\textrm{F}}\,\mathrm{\Pi }\,\textbf{I}, \end{array} \end{aligned}$$

where $\lambda ^{\alpha }$ and $\mu ^{\alpha }$ represent the Lamé constants for the solid phases.

By evaluating the dissipative momentum production, the restrictions are derived as follows:

21 $$\begin{aligned} \begin{array}{lcll} {\hat{{\textbf {p}}}}^{\textrm{F}}\,&{}=&{}\,\textrm{grad}\,{\textrm{n}^{\textrm{F}}}\,\lambda \,+\,\mathrm{\Pi }\,\textrm{grad}\,{\textrm{n}^{\textrm{F}}}\,+\,\gamma ^\textrm{F}_{\textbf{w}_{\textrm{F}\beta \textrm{S}}}\,\textbf{w}_{\textrm{F}\beta \textrm{S}}\, \\ {} \quad&{}-&{}\,\gamma ^\textrm{F}_{\textbf{w}_{\textrm{FS}}}\,\textbf{w}_{\textrm{FS}}\\ {\hat{{\textbf { p}}}}^{\textrm{F}\beta }\,&{}=&{}\,\quad-\mathrm{\Pi }\,\textrm{grad}\,{\textrm{n}^{\textrm{F}}}\,-\,\gamma ^\textrm{F}_{\textbf{w}_{\textrm{F}\beta \textrm{S}}}\,\textbf{w}_{\textrm{F}\beta \textrm{S}}\,-\,\gamma ^\textrm{F}_{\textbf{w}_{\textrm{FS}}}\,\textbf{w}_{\textrm{FS}}. \\ \end{array} \end{aligned}$$

By further evaluating the dissipative momentum production together with the balance of momentum for fluid components, the seepage velocity of the external components results in

22 $$\textbf{w}_{\textrm{F}\beta \textrm{S}}\,=\,\displaystyle \frac{\textrm{1}}{\gamma ^\textrm{F}_{\textbf{w}_{\textrm{F}\beta \textrm{S}}}}\,(\textrm{n}^{\textrm{F}}\,\textrm{grad}\, \mathrm{\Pi }\,-\,\gamma ^\textrm{F}_{\textbf{w}_{\textrm{FS}}}\,\textbf{w}_{\textrm{FS}}).$$

The seepage velocity for the fluid and therefore, the advective part is evaluated analogs to the seepage velocity of external components using the balance of momentum, which leads to

23 $$\begin{aligned} \textrm{n}^{\textrm{F}}\textbf{w}_{\textrm{FS}}= \textbf{K}_{\textrm{F}} \, (-\textrm{grad}\,\lambda ). \end{aligned}$$

A detailed derivation of the restriction for stresses, dissipative momentum production and seepage velocities can be found in Section 7.3 of the appendix.

## Microscale

On the microscale, several metabolic processes take place consisting of fat metabolism, e.g., oxidation of FFA or synthesis of TG, and glucose metabolism, e.g., glycolysis of glucose. Further information about the glucose metabolism is provided in Ricken et al. (2015) and König et al. (2012), information on the fat metabolism in Schleicher et al. (2017).

**Fig. 5:**
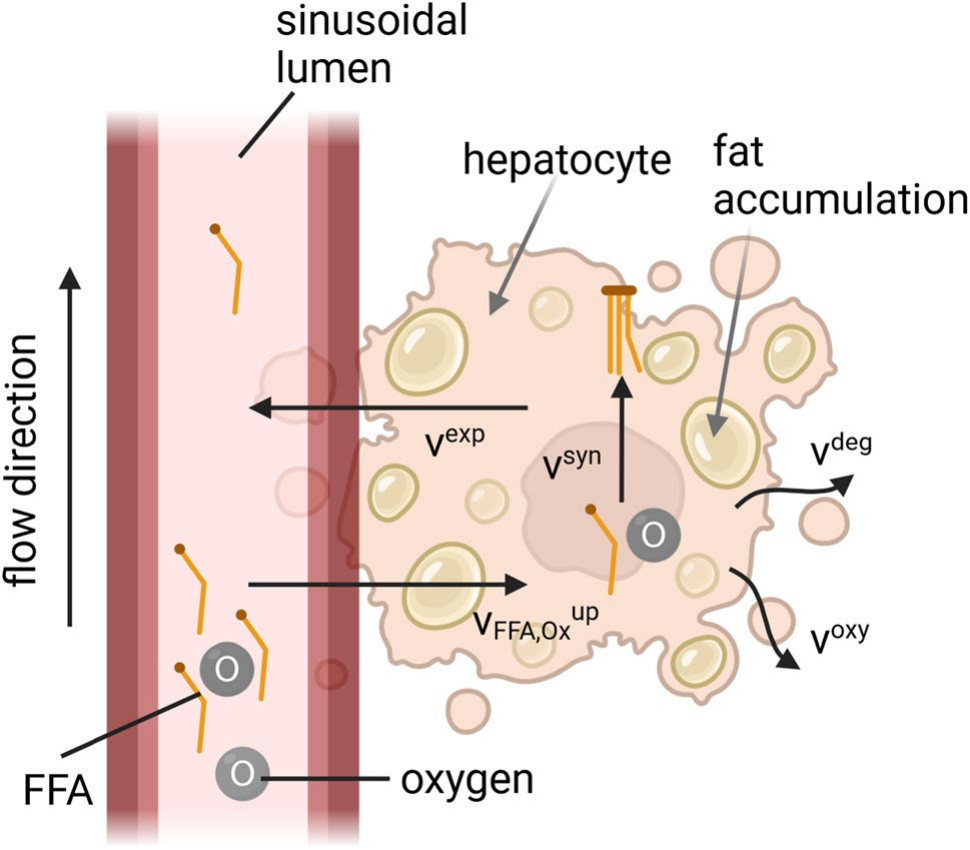
Outline of the main aspects of the hepatic fat metabolism described by a mathematical model in Schleicher et al. (2017). Key metabolic processes in the model are the uptake of plasma FFAs and oxygen ($\rm v_\text{FFA, Ox}^\text{up}$), oxidative processes $\rm v_\text{oxy}$, the synthesis ($\rm v^\text{syn}$), export ($\rm v^\text{exp}$), degradation ($\rm v^\text{deg}$) and the secretion of TGs. The model fits the condition of the perfusion model on the macroscale, delivering external flow conditions

Fatty liver disease is characterized by an inhomogeneous distribution of TG along the liver lobules. Schleicher et al. (2017) developed a mathematical model in form of an ODE system which we adapted for inclusion in our perfusion model based on TPM. The set of ODEs is embedded in the macro-model with dependencies on actual concentration solutions ($\textrm{c}^{\alpha \beta }$) and volume fractions ($\textrm{n}^{\alpha }$) from the lobule scale in each Gauss point, see Waschinsky et al. (2016, 2017). This delivers a spatial, perfusion-dependent evaluation of the nutrient deposition and the fat distribution in the cells. The ODE model involves the uptake mechanism of plasma FFAs and oxygen, oxidative processes, the synthesis as well as the secretion of TGs (as depicted in Fig. 5). In the following, the reactions of the main solutes are summarized according to Schleicher et al. (2017):

24 $$\begin{aligned} \begin{array}{lll} {\textrm{c}_{\textrm{FFA}}^{\textrm{int}}}\,&{}=&{}\,{\textrm{v}_{\mathrm{{FFA}}}^{\textrm{up}}} -\,\textrm{v}^{\textrm{syn}} -\,\textrm{v}^{\textrm{oxy}} \\ {\textrm{c}_{\textrm{Ox}}^{\textrm{int}}}\,&{}=&{}\,{\textrm{v}_{\textrm{Ox}}^{\textrm{up}}} -\,\textrm{v}^{\textrm{oxy}}-\,\textrm{v}^{\textrm{deg}}, \end{array} \end{aligned}$$

where ${\textrm{c}_{\textrm{FFA}}^{\textrm{int}}}$ and ${\textrm{c}_{\textrm{Ox}}^{\textrm{int}}}$ describe the intercellular concentrations of FFA and oxygen in the hepatocytes. The concentrations depend on the uptake processes ${\textrm{v}_{\mathrm{{FFA}}}^{\textrm{up}}}$ and ${\textrm{v}_{\mathrm{{Ox}}}^{\textrm{up}}}$, which were realized by Michaelis–Menten kinetics depending on parameters $\mathrm v_{\rm max}$ and $\rm K_\text M$. FFAs are esterified to TG, conversely the synthesis rate $\textrm{v}^{\textrm{syn}}$ of TGs influences the intercellular concentration of FFAs. Mitochondrial FFA oxidation $\textrm{v}^{\textrm{oxy}}$ consumes FFAs as well as oxygen. The intercellular oxygen concentration is not only responsible for FFA oxidation, but it is also involved in other oxidative processes such as glucose oxidation. This usage of oxygen is described in $\textrm{v}^{\textrm{deg}}$. Detailed information about the equations describing the fat metabolism can be found in Schleicher et al. (2017). The esterification of FFAs to TGs depends on the synthesis rate $\rm v^\text{syn}$ and additionally on the export rate $\textrm{v}^{\textrm{exp}}$, representing hepatic lipid dynamics ($\hat{\rho }^{\textrm{T}}$)

25 $$\begin{aligned} \hat{\rho }^{\textrm{T}}\,=\,\textrm{v}^{\textrm{syn}}-\,\textrm{v}^{\textrm{exp}}. \end{aligned}$$

Fig. 5 summarizes the key metabolic reactions and metabolite concentrations. We use inflow boundary conditions to provide the plasma concentrations of FFA and oxygen. Here, the chemical potential is calculated as a degree of freedom, and subsequently, the resulting concentration is determined. The inflow and outflow boundary conditions are applied by the initial chemical potential of the corresponding concentration; a concentration gradient is created by different values of inflow and outflow. The dependencies on the microscale affect the establishment of an FFA gradient as well as an oxygen gradient on the lobule scale. Since oxygen is an important factor for fatty acid oxidation, an oxygen gradient may determine the zonation pattern of fat accumulation, as fat storage is increased in areas with a low oxygen concentration (Chen et al. 2019). The gradients of the plasma concentrations of oxygen and FFA depend on the blood motion of the carrier phase (advection) and on the osmotic pressure (diffusion), see Section 2.5.

To couple the macroscale and the microscale, we assume a huge ratio between the length of both scales. We use the concept of representative volume elements (RVE), see Ricken et al. (2015), which leads to the connection

26 $$\begin{aligned} \{\cdot \} = \frac{1}{\textrm{V}} \int \limits _{\partial \textrm{B}_{\textrm{S}}} \overline{\{\cdot \}} \, \textrm{dv}, \end{aligned}$$

where $\{\cdot \}$ represents the scalar quantities on the macroscale and $\overline{\{\cdot \}}$ shows the scalar quantities on the microscale. This leads to the description of the rates

27 $$\begin{aligned} \begin{aligned}&\hat{\textrm{c}}^\textrm{FFA} = \frac{1}{\textrm{V}} \int \limits _{\partial \textrm{B}_{\textrm{S}}} \overline{\hat{\textrm{c}}^{\textrm{FFA}}} \, \textrm{dv},\hspace{1cm} \hat{\textrm{c}}^{\textrm{Ox}} = \frac{1}{\textrm{V}} \int \limits _{\partial \textrm{B}_{\textrm{S}}} \overline{\hat{\textrm{c}}^{\textrm{Ox}}} \, \textrm{dv}, \, \\&\hat{{\rho }}^{\textrm{T}} = \frac{1}{\textrm{V}}\int \limits _{\partial \textrm{B}_{\textrm{S}}} \overline{\hat{{\rho }}^{\textrm{T}}} \, \textrm{dv}. \end{aligned} \end{aligned}$$

Regarding these dependencies, the ODE model is solved in each Gauss point by computing the concentration change for FFAs ($\hat{\textrm{c}}^\textrm{FFA}$) and oxygen ($\hat{\textrm{c}}^{\textrm{Ox}}$), as well as the rate for the fat accumulation $\hat{{\rho }}^{\textrm{T}}$.

## Numerical solution

The previously introduced material formulation for the multiphase and multicomponent liver model leads to a reduction of the unknown quantities and a set of coupled differential equations. A numerical approximation, the Finite Element Method (FEM), is applied for the numerical solution of the macroscopic field equations. In order to close the system of partial differential equations, the unknown quantities are solved by governing equations. This approach is used to solve the following set of degrees of freedom:

28 $$\begin{aligned} \mathcal {R}=[\textbf{u}_{\textrm{S}},\,\text {p}^{\text {FR}},\,\textrm{n}^{\textrm{S}},\,\textrm{n}^{\textrm{T}},\,\mu ^{\alpha \beta }]. \end{aligned}$$

The solution of the displacement $\textbf{u}_{\textrm{S}}$ is calculated by the evaluation of the balance of momentum for the mixture. The Lagrange parameter $\lambda$ can be identified as the hydrostatic pressure $\text {p}^{\text {FR}}$, cf. Boer (2000). The hydrostatic pressure is solved using the balance of mass for the mixture of the phases. Furthermore, applying the balance of mass for the single tissue phases of solid and fat leads to the solution of the volume fractions $\textrm{n}^{\textrm{S}}$ and $\textrm{n}^{\textrm{T}}$ and the balance of mass for the solutes solves the chemical potential $\mu ^{\alpha \beta }$ of the solutes. The set of unknown quantities is determined via the Galerkin procedure, where the balance equations are transformed to their weak form and weighted by the independent test functions $\delta \textbf{u}_{\textrm{S}},\,\delta {\textrm{p}}^{\textrm{FR}},\,\delta \textrm{n}^{\textrm{S}},\,\delta \textrm{n}^{\textrm{T}},\,\delta \mu ^{\alpha \beta }$. The relevant weak formulations in the reference placement are introduced according to Werner (2017) and Lambers (2023) as follows:

- Balance of momentum for the mixture of phases:
  29 $$ \rm G^{\text M}_\text {Mom} = \text {div} (\textbf{T}^{\textrm{S}}+ \textbf{T}^{\textrm{T}}+ \textbf{T}^{\textrm{F}}+ \textbf{T}^{\alpha \beta }), $$
  which results in the weak form
  30 $$\begin{aligned} \begin{aligned} \displaystyle {\int \limits _{\mathrm{B_{0S}}}}\,\left\{ \sum _{\alpha =\textrm{1}}^{(\textrm{S,T,F},\alpha \beta )}\,\textbf{P}^{\alpha }\,\textrm{grad}\,\delta \textbf{u}_{\textrm{S}}\,\right\} \,\textrm{dV}\, \\ =\,\int \limits _{\partial \mathrm{B_{0S}}}\,\left\{ \textbf{t}_\textrm{0}\,\delta \textbf{u}_{\textrm{S}}\right\} \,\textrm{dA}. \end{aligned} \end{aligned}$$
- Balance of mass for the mixture of phases:
  31 $$\begin{aligned} \begin{aligned} \mathrm G^{\text{M}}_{\text{Mass}} = \,&(\textrm{n}^{\textrm{S}})^{\prime }_{\textrm{S}}+ \textrm{n}^{\textrm{S}}\, {\text{div}} \, \textbf{x}^{\prime }_{\textrm{S}}+ (\textrm{n}^{\textrm{T}})^{\prime }_{\textrm{S}}+ \textrm{n}^{\textrm{T}}\, {\text{div}} \, \textbf{x}^{\prime }_{\textrm{S}}\\ {}&- \frac{\hat{\rho }^{\textrm{T}}}{\rho ^{\textrm{TR}}} + (\textrm{n}^{\textrm{F}})^{\prime }_{\textrm{F}}+ \textrm{n}^{\textrm{F}}\, {\text{div}} \, \textbf{x}^{\prime }_{\textrm{F}}, \end{aligned} \end{aligned}$$
  with the resulting weak form
  32 $$  \int_{{\text{B}}_{{\text{0S}}} } {\left\{ {{\text{J}}_{\text{S}} {\text{n}}^{\text{F}} {\bf{w}}_{FS} {\bf{F}}_S^{T - 1} {\text{Grad}}\delta {\text{p}}^{{\text{FR}}} + {\text{J}}_{\text{S}} {\text{tr}}\;{\text{D}_{{S}}} \delta {\text{p}}^{{\text{FR}}}  - {\text{J}}_{\text{S}} \hat{\rho }^{\text{T}} \frac{1}{{\rho ^{{\text{TR}}} }}\delta {\text{p}}^{{\text{FR}}} {\text{J}}_{\text{S}} {\bf{F}}_{\text{S}}^{T - 1} {\text{n}}^{\text{F}} {\bf{w}}_{{\text{FS}}} \cdot {\bf{n}}\delta {\text{p}}^{{\text{FR}}} } \right\}} {\text{dA}}  $$
- Balances of mass for the liver-tissue and the fat development:
  33 $$\begin{gathered} \int_{B_{0S} } {\left\{ {{\text{J}}_{\text{S}} ({\text{n}}^{\text{S}} )_{\text{S}}^{\prime} \delta {\text{n}}^{\text{S}} + {\text{n}}^{\text{S}} \, {\text{J}}_{\text{S}} \, {\text{tr}} \, {\mathbf{D}}_{\text{S}} \, \delta {\text{n}}^{\text{S}} } \right\}{\text{dV}} = 0} \hfill \\  \int_{B_{0S} } {\left\{ {{\text{J}}_{\text{S}} \, ({\text{n}}^{\text{T}} )_{\text{S}}^{\prime} \delta {\text{n}}^{\text{T}} + {\text{n}}^{\text{T}}\,  {\text{J}}_{\text{S}} \, {\text{tr}}\,{\mathbf{D}}_{\text{S}} \, \delta {\text{n}}^{\text{T}} {\text{J}}_{\text{S}} \frac{{\hat{\rho }^{\text{T}} }}{{\rho ^{{\text{TR}}} }}\delta {\text{n}}^{\text{T}} } \right\}{\text{dV}} = 0} \hfill \\ \end{gathered}$$
- Balance of mass for the solutes in the tissue phase (no transport of solutes):
  34 $$\begin{aligned} \begin{aligned} \displaystyle {\int \limits _{\mathrm{B_{0S}}}}\,&\left\{ \textrm{J}_{\textrm{S}}\,\textrm{n}^{\textrm{S}}\,(\textrm{c}^\mathrm{S\beta })'_{\textrm{S}}\,\delta \mu ^{\textrm{S}\beta }\,+\textrm{J}_{\textrm{S}}\,\textrm{c}^\mathrm{S\beta }\,(\textrm{n}^{\textrm{S}})^{\prime }_{\textrm{S}}\,\delta \mu ^{\textrm{S}\beta }\, \right. \\ {}&+ \left. \textrm{J}_{\textrm{S}}\,\textrm{n}^{\textrm{S}}\,\textrm{c}^\mathrm{S\beta }\,\textrm{div}\,\textbf{x}^{\prime }_{\textrm{S}}\,\delta \mu ^{\textrm{S}\beta }\,-\,\textrm{J}_{\textrm{S}}\,\frac{\hat{\rho }^{\textrm{S}\beta }}{\textrm{M}^{\beta }_\textrm{mol}}\,\delta \mu ^{\textrm{S}\beta }\right\} \,\textrm{dV}\,\\ {}&=\textrm{0}. \end{aligned} \end{aligned}$$
- Balance of mass for the solutes in the fluid phase:
  35 $$\begin{aligned} \begin{aligned} \displaystyle {\int \limits _{\mathrm{B_{0S}}}}&\left\{ \textrm{J}_{\textrm{S}}\,\textrm{n}^{\textrm{F}}\,(\textrm{c}^\mathrm{F\beta })'_{\textrm{S}}\,\delta \mu ^{\textrm{F}\beta }\,+\textrm{J}_{\textrm{S}}\,\textrm{c}^\mathrm{F\beta }\,(\textrm{n}^{\textrm{F}})^{\prime }_{\textrm{S}}\,\delta \mu ^{\textrm{F}\beta }\, \right. \\&\left. -\,\textrm{J}_{\textrm{S}}\,(\textrm{n}^{\textrm{F}}\,\textrm{c}^\mathrm{F\beta }\,\textbf{w}_{\textrm{F}\beta \textrm{S}})\,\cdot \,\textrm{grad} \, \delta \mu ^{\textrm{F}\beta }\,\right\} \,\textrm{dV}\, +\\&\left\{ \,\textrm{J}_{\textrm{S}}\,\textrm{n}^{\textrm{F}}\,\textrm{c}^\mathrm{F\beta }\,\textrm{div} \, \textbf{x}^{\prime }_{\textrm{S}}\,\delta \mu ^{\textrm{F}\beta }\,-\,\textrm{J}_{\textrm{S}}\,\displaystyle \frac{\hat{\rho }^{\textrm{F}\beta }}{\textrm{M}^{\beta }_\textrm{mol}}\,\delta \mu ^{\textrm{F}\beta }\right\} \,\textrm{dV}\, \\&=\,\displaystyle {\int \limits _{\partial \mathrm{B_{0S}}}}\,\left\{ \textrm{J}_{\textrm{S}}\,\textrm{n}^{\textrm{F}}\,\textrm{c}^\mathrm{F\beta }\,\textbf{w}_{\textrm{F}\beta \textrm{S}}\,\cdot \,\mathrm{{\textbf {n}}}\,\delta \mu ^{\textrm{F}\beta }\right\} \,\textrm{dA}. \end{aligned} \end{aligned}$$

A stable numerical evaluation is guaranteed by using Taylor-Hood elements, which have quadratic ansatz functions for the displacement $\textbf{u}_{\textrm{S}}$ and linear ansatz functions for the pressure $\text {p}^{\text {FR}}$, the volume factions $\textrm{n}^{\textrm{S}},\,\textrm{n}^{\textrm{T}}$ as well as the chemical potential $\mu ^{\alpha \beta }$.

**Table 1:** Material parameters of a liver lobule

Parameter	Value	Unit	Remark
$\textrm{n}^{\textrm{S}}_{\textrm{0S}}$	0.8	–	Initial volume fraction solid, cf. Werner (2017)
$\textrm{n}^{\textrm{T}}_{\textrm{0S}}$	0.02	–	Initial volume fraction fat, cf. Werner (2017)
$\textrm{n}^{\textrm{F}}_{\textrm{0S}}$	0.18	–	Initial volume fraction fluid, cf. Werner (2017)
$\mu ^{\textrm{S}},\,\mu ^{\textrm{T}}$	$4\,\cdot \,10^{4}$	Pa	Lamé constant, cf. George et al. (2018), Evans et al. (2013)
$\lambda ^{\textrm{S}},\,\lambda ^{\textrm{T}}$	$3\,\cdot \,10^{4}$	Pa	Lamé constant, cf. George et al. (2018), Evans et al. (2013)
$\theta$	280	K	Temperature
$\textrm{R}$	8.3144	J/molK	Gas constant
$\textrm{k}_{\textrm{D}}$	$4.5\,\cdot \,10^{-10}$	m/s	Darcy coefficient

## In-silico study

In this work, different boundary conditions are examined by changing the inflow and outflow of blood and their influences on the liver perfusion are illustrated. Here, the previously derived equations were implemented and solved in a user element of the finite element program FEAP Taylor (2012). A time discretization is performed with the Newmark method. Furthermore, we used Taylor-Hood elements Taylor and Hood (1973) with quadratic ansatz functions for the displacement and linear ansatz functions for the remaining field quantities. Thereby, the calculations are two-dimensional in the plane of the lobules of the liver. A later extension of the processes to the third dimension is possible due to the derivation, but not foreseen in this study, since the processes to be investigated are to be examined only in the lobular plane and a three dimensional simulation would require significantly more computational power.

**Fig. 6:**
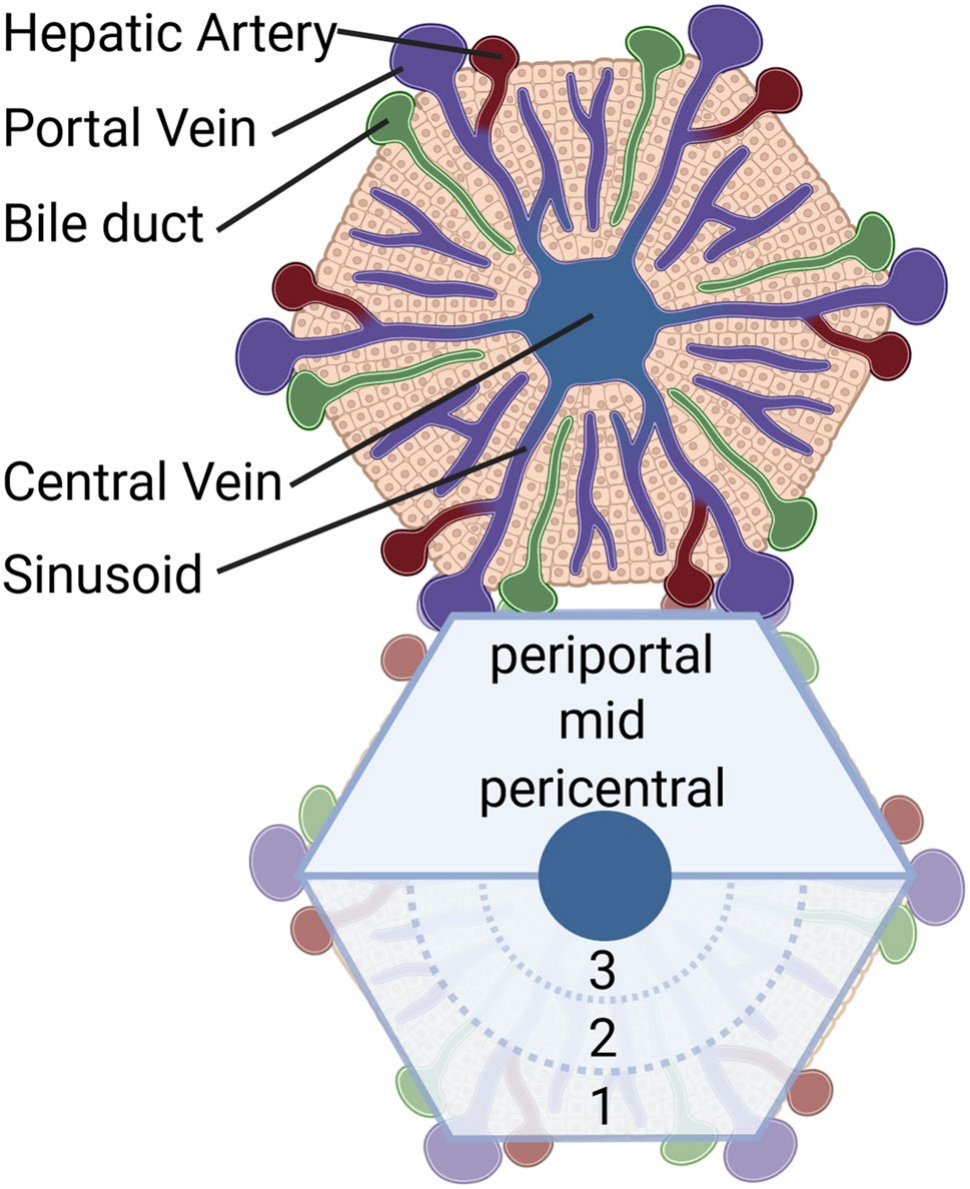
Composition of hepatic lobular scale (left) with division into characteristic zones, namely zone 1, 2 and 3, as well as a periportal, mid or pericentral zone

In the first step, we want to perform a verification of the coupled function-perfusion model on lobular and cell scale with experimentally observed fat distributions based on histological images. In a second step, we investigate how the boundary conditions of the fluid pressure influence the fat metabolism and distribution. In addition to the spatial accumulation of fat, the distribution of free fatty acids and oxygen is also investigated, as these substances are primarily responsible for fat metabolism. In Ricken et al. (2018), we have observed a strong influence of the inflow boundary condition on the distribution of fat along the liver lobule. We have analyzed the influence of the inflow of portal blood on the solute distribution solely through the portal triad as opposed to through additional venules on the periphery of the lobules. We have found an agreement of the solute patterns between the numerical simulation with additional venules and experiments. Schleicher et al. (2017) observed a connection between the oxygen gradient within the lobule and the fat zonation pattern, which indicates that the pericentral fat deposition is connected to a low supply of oxygenated blood to the hepatocytes in the area around the central vein. The liver lobules are divided into three zones, which are oriented according to the distance to the inflowing blood at the portal fields. These zones are either declared numerically (Zone 1 - Zone 3) or according to their location in the lobule as periportal, mid or pericentral zone, see also Fig. 6.

Since there is a high influence of the microperfusion on the hepatic fat metabolism, we want to understand the consequences of outflow obstruction on the fat accumulation in a further step. Based on the mentioned knowledge of the inflow conditions and the zonation of fat accumulation, we apply different boundary conditions as well as an oxygen gradient from the portal triad to the central vein, which has an influence on the fat metabolism on the cellular scale.

### Boundary conditions and material parameters

**Fig. 7:**
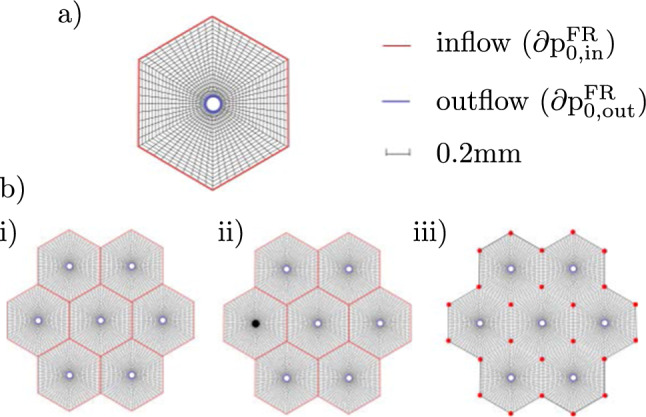
Pressure boundary conditions for three different situations: (**a**) one idealized liver lobule; (**b**) for a group of seven idealized lobules and the following conditions: (i) undisturbed pressure conditions, (ii) outflow obstruction in one liver lobule, (iii) outflow obstruction and connection with neighboring lobules. Red lines and dots represent inflow conditions with the initial value $\partial \text {p}^{\text {FR}}_{\textrm{0, in}}$. Blue lines show the outflow conditions at the central vein with the initial pressure $\partial \text {p}^{\text {FR}}_{\textrm{0, out}}.$

For the validation of the results, a single lobule is considered, shown in Fig. 7a. The red lines represent the inflow and the blue ones the outflow area. As discussed in Ricken et al. (2018), the inflow area consists of the portal triad and additional venules, which are positioned on the lobule periphery. Although the portal fields are located at the corners of the lobule, the blood is distributed over the lobule after entry so that the entire tissue is perfused. We apply constant values of external FFA and oxygen concentrations as inflow conditions and fixed displacements on the in- and outflow nodes of the FE mesh.

The illustrative example is performed on the geometry of a group of lobules using the material parameters shown in Table 1. These parameters are taken from the literature and can be obtained, for example, via Magnetic Resonance Imaging, cf., e.g., Seyedpour et al. (2021a). Since the exact structure of microperfusion and blood inflow in the lobules is not fully understood, we aim to investigate different pressure boundary conditions and thus different microperfusions in the group of lobules. Figure 7b) shows the FE mesh of the group with three different boundary conditions as an extension of former studies, cf. Lambers et al. (2018).

The inflow and outflow conditions of each example are again marked by red and blue lines, respectively. Here, boundary condition i) represents the classical lobular condition, ii) shows unchanged inflow but an obstructed outflow in one lobule and iii) illustrates changed inflow conditions with undisturbed flow between the neighboring lobules. Further details on the applied boundary conditions can be found in Lambers (2023).

### Model verification

Dependent on the initial and boundary conditions of concentrations, the applied metabolic model on the cellular scale results in a zonated accumulation of fat.

**Fig. 8:**
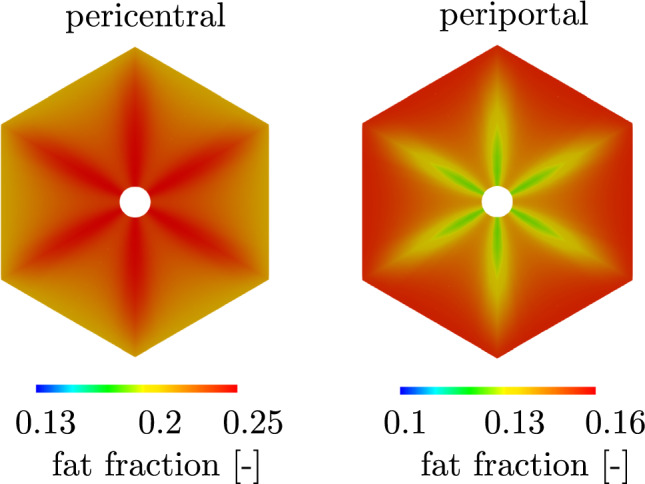
(**a**) periportal zonation of fat accumulation, (**b**) pericentral zonation of fat accumulation

**Fig. 9:**
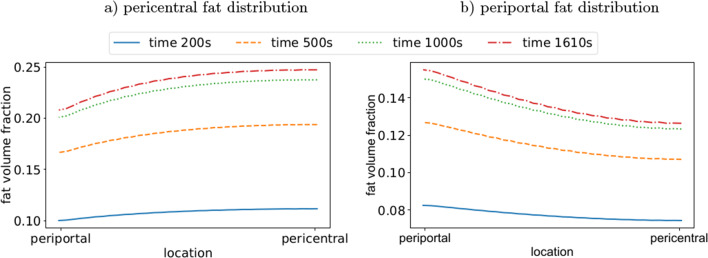
Distribution of fat fraction at different locations along the liver lobule. Fat content is evaluated after 200, 500, 1000 and 1610 s during (**a**) pericentral zonation of fat accumulation, (**b**) periportal zonation of fat accumulation

**Fig. 10:**
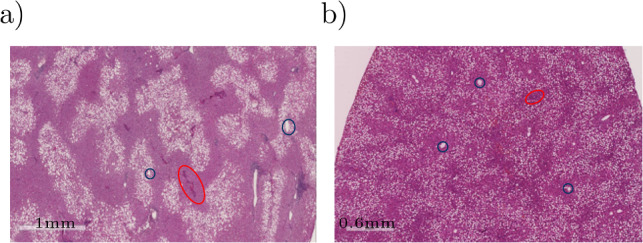
Zonation patterns of fat accumulation. (**a**) pericentral zonation of fat accumulation in a steatotic human liver, (**b**) periportal zonation of fat accumulation in a steatotic rat liver. Exemplary central veins are marked in blue, portal fields in red

The results in Fig. 8 show the fat accumulation in a single liver lobule for different initial concentrations of FFA (3 mmol/l and 0.2 mmol/l) and an oxygen concentration of 0.091 mmol/l. The spatial distribution shows a clearly zonated pattern with a higher accumulation either in the periportal or pericentral zone of the lobule. A high intake of FFA combined with a low oxygen concentration results in increased formation of fat in the hepatocytes near the central vein (see Fig. 9a). This pericentral fat zonation pattern is often observed in human MASLD where fat-laden hepatocytes are located near the central vein (Kleiner and Makhlouf 2016). In contrast, reducing the intake of FFAs changes the zonation pattern to an accumulation fat-laden hepatocytes of steatosis at the outer edges of a liver lobule, i.e., a periportal zonation pattern of fat, which is shown in Fig. 9b. However, also the periportal zonation of MASLD is associated with fat accumulation in liver lobules, cf. Liu et al. (2016). For a verification, H&E-stained histological images obtained experimentally and clinically (ethical vote: UKJ_2018_1246_material) are used. The histological image in Fig. 10a demonstrates a pericentral zonation pattern in a human liver with moderate-severity steatosis. The fat-laden hepatocytes are located around the central veins, with less fat-containing hepatocytes in the periportal zone. In contrast, in Fig. 10b, a section displaying periportal fat accumulation is depicted. Here, fat-laden hepatocytes with white, round fat vacuoles appear primarily in the periportal region. The central veins remain devoid of fat, rendering them clearly visible. The comparison of the observed fat patterns and the simulation results indicates that zonated fat accumulation can be accurately represented using the coupled continuum biomechanical model. However, the simulation results show a symmetrical zonation pattern due to the idealized symmetrical geometry. The zonation patterns in the liver lobules are more irregular, which can be explained by the heterogeneity in the geometries. Since a pericentral zonation is mostly observed in human MASLD, the following evaluations will be examined with this zonation pattern.

### Evaluation of the microperfusion

**Fig. 11:**
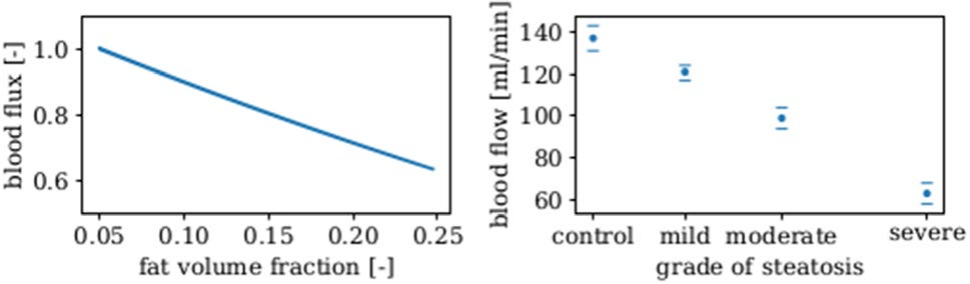
Dependency of blood flow on fat accumulation. (**a**) Numerical simulation of the relation between the fat accumulation $\textrm{n}^{\textrm{T}}$ [-] and the relative reduction of the seepage velocity $\textbf{w}_{\textrm{FS}}$ [-] with respect to the initial velocity. The diagram shows the results over 1610 s of simulation time at the mid zone of a lobule. (**b**) Similar results were shown in a study of an animal model which evaluates the *in vivo* effect of steatosis on hepatic blood flow, see Seifalian et al. (1999).

In the next example, we investigate the association of fat content and blood perfusion. In Fig. 11, we illustrate the impact of the accumulation of fat on the microperfusion in one lobule.

In detail, we focus on the seepage velocity in relation to the fat volume. The increase in fat leads to the reduction of the seepage velocity, the relation is nearly linear. These results are in good agreement with experimental data from Seifalian et al. (1999) which showed similar results for the effect of graded steatosis on the blood flow in the microcirculation.

**Fig. 12:**
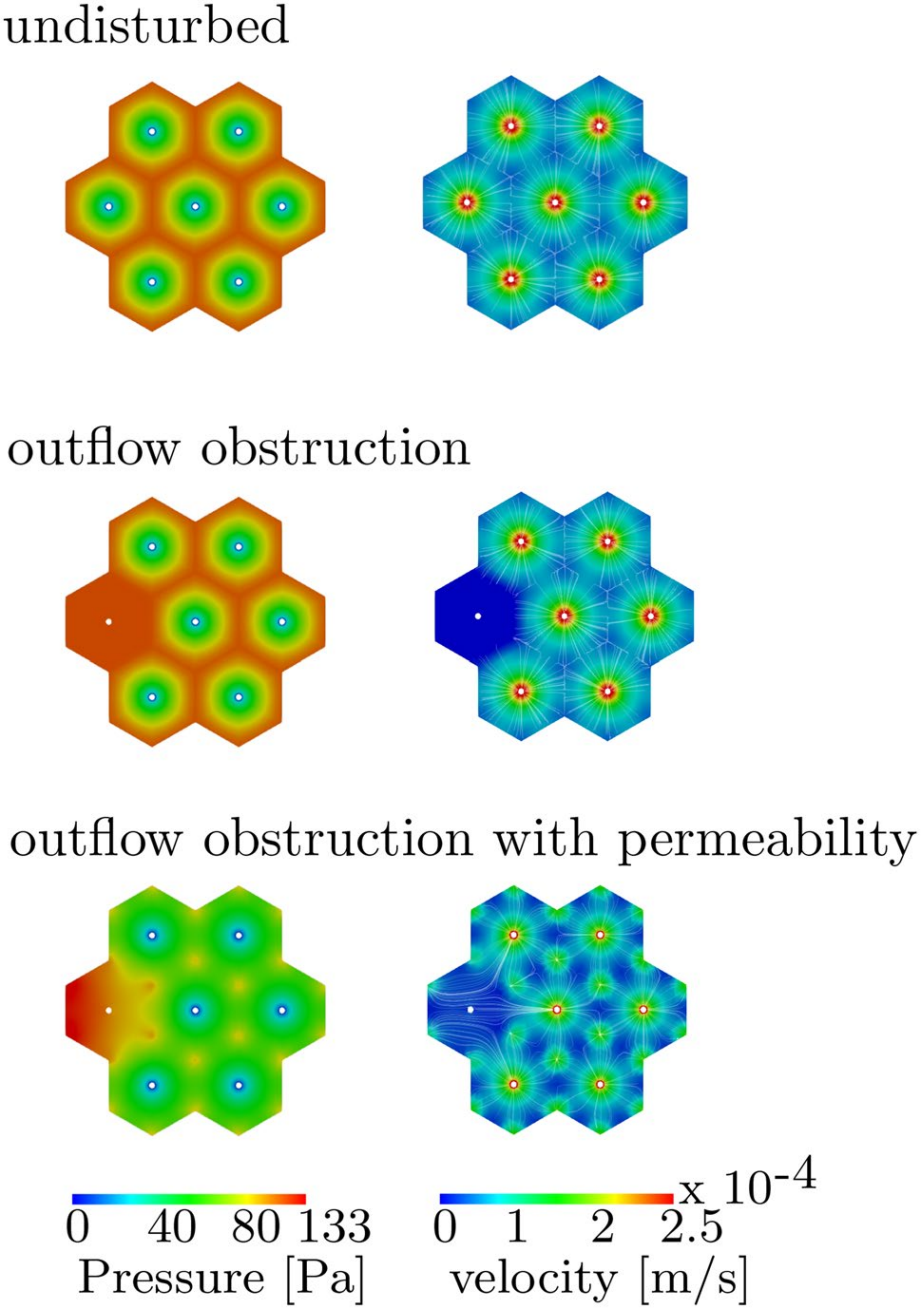
Left hand side: contour plot for the pressure distribution [$\frac{\textrm{N}}{\textrm{m}^{2}}$]. The estimated pressure difference ($\sim$133 Pa) is between inflow and outflow areas; Right hand side: contour plot for velocity distribution [$\frac{\textrm{m}}{\textrm{s}}$]. Streamlines denote the preferred flow direction for the stationary solution using anisotropic permeability. (**a**) Undisturbed distribution, (**b**) Outflow obstruction, (**c**) Outflow obstruction with permeability

In Fig. 12, we evaluate the microperfusion for the three applied boundary conditions in a group of liver lobules. The pressure is illustrated on the left and the seepage velocity is depicted on the right. The decisive factors affecting the results for the pressure pattern are the applied boundary conditions stated as Dirichlet values for the in- and outflow. We assume a pressure difference of about 133 Pa respectively 1 mmHg between the inflow and outflow areas. The applied Darcy formulation of the blood is characterized by a seepage velocity, which is proportional to the pressure gradient.

**Fig. 13:**
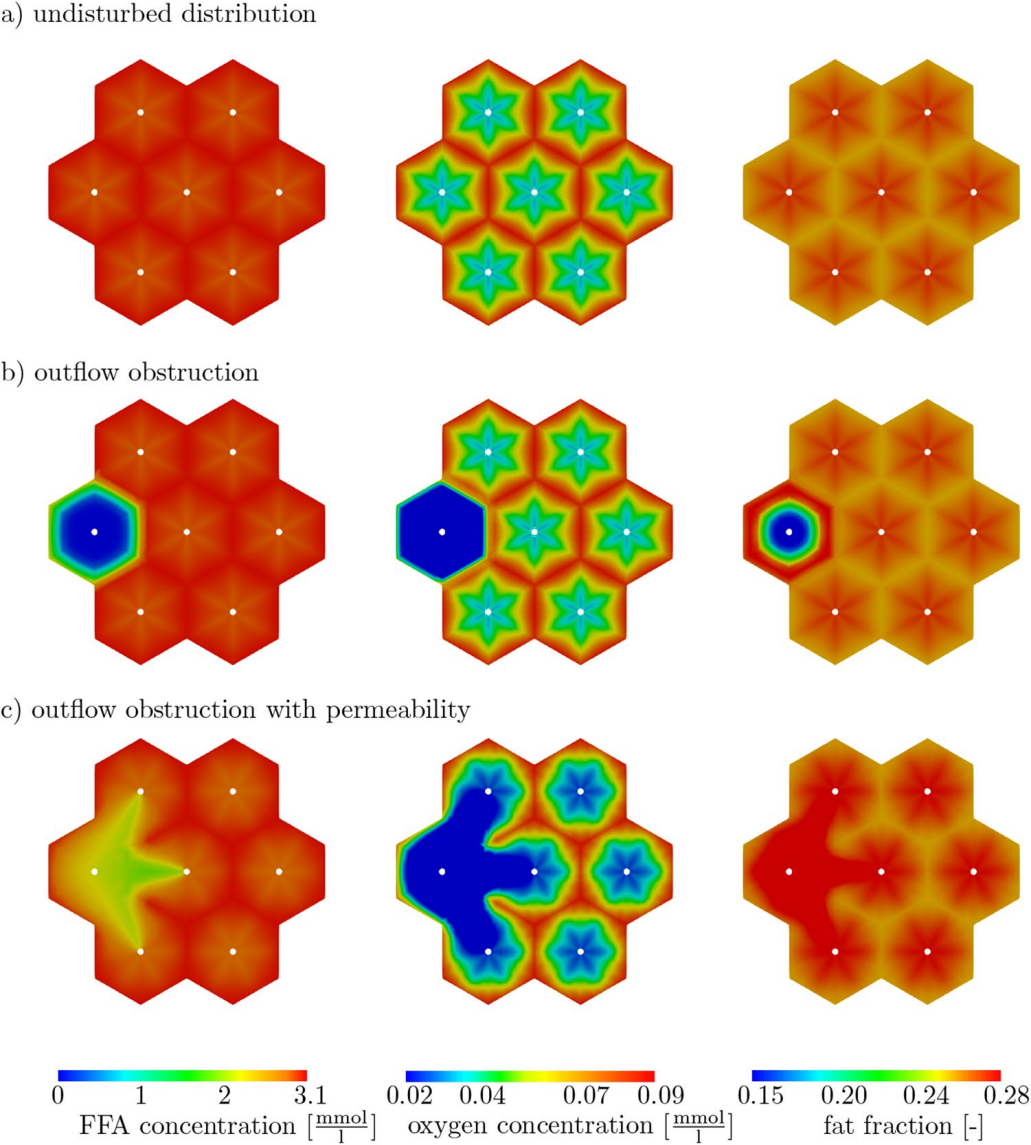
Deposition of nutrients after 1610 s of simulation time. First column: contour plot for FFA concentration [$\frac{\textrm{mmol}}{\textrm{l}}$]. Second column: contour plot for oxygen concentration [$\frac{\textrm{mmol}}{\textrm{l}}$]. Third column: contour plot for fat fraction [-]. (**a**) Undisturbed distribution, (**b**) Outflow obstruction, (**c**) Outflow obstruction with permeability

The flow direction is controlled by applied pressure gradients from inflow to outflow areas. Besides the seepage velocity, the flux direction is evaluated by streamlines, which denote the preferred flow direction for the stationary solution. Apart from the applied pressure boundary conditions, the permeability of the lobules has the greatest influence on the seepage velocity. Three different scenarios were simulated: (a) The first assumption is that the lobules have pericentral pressure gradients and contains an unimpaired blood flow from the lobule periphery to the central vein. (b) For the second condition, we apply an outflow obstruction on the left lobule. No pressure gradient exists in the obstructed area, as the left lobule has only inflow conditions with an applied pressure of 100 $\frac{\textrm{N}}{\textrm{m}^{2}}$ on the periphery of the lobules. The microperfusion is therefore slowed down and there is no possibility for advection. (c) Finally, we assume a more physiological scenario in the third condition, where the flux of the obstructed lobule can be rearranged and is directed to the neighboring lobules. This is ensured by applying the pressure only in the portal triad, yielding to a pressure gradient from the left lobule to the adjacent outflow areas. The pressure on the inflow is increased by 30 percent in order to speed up the flux velocity. The streamlines show the drain of the flux to the neighboring lobules. In this study we only focus on idealized geometries of liver lobules and the resulting microperfusion, however an evaluation of microperfusion depending on realistic geometries can be found in Lambers (2023).

### Evaluation of fat metabolism

Due to the different boundary conditions of the microperfusion, a varied evaluation of the fat metabolism is achieved, cf. Lambers (2023). In the first two columns in Fig. 13, we depict the external concentration of FFA and oxygen. In the last column we illustrate the volume fraction of fat. The transport of external concentration depends on advection and diffusion. The advection describes the transport of the solutes via the velocity of the blood; whereas, diffusion is characterized by a steady movement from high-concentration areas to low-concentration areas. Hence, the transport of FFA and oxygen depends on the periportal pressure gradient (advection) and on periportal external concentration gradients (diffusion). Furthermore, the fat accumulation, which is a chemical conversion process, influences the decomposition rate of substances in the lobule. In the first example (a), we see external gradients of FFA and oxygen with a higher concentration at the inflow area and a lower one at the center of the lobule. These concentration gradients in the lobule arise due to the changed transport via the blood with an increased seepage velocity at the central vein (outflow) and because of the altered diffusion conditions. Additionally, the chemical processes lead to a reduction of the external concentrations of FFA as they esterify to fat. Both periportal external gradients, FFA and oxygen, are important for the fat metabolism, even though they work against each other, so that a high concentration of FFA results in an increased esterification to fat, whereas a high concentration of oxygen decreases the fat deposition due to an increase in the rate of fatty acid oxidation. Schleicher et al. (2017) observed a high influence of the oxygen gradient on pericentral fat accumulation. Such a zonated fat deposition is usually observed during a high-fat diet and is evoked by lower oxygen values at the central vein, so that fatty acid oxidation is reduced and the accumulation of fat is increased. The second example (b) shows the importance of the microperfusion, which is clearly reduced in the left lobule. There is no pressure gradient, and in consequence no advection, which encourages the concentration transport. The diffusion leads to a smooth compensation of the concentrations, but the effect is insufficient to deliver external metabolites to the center of the lobulus. Furthermore, the absence of external metabolites results in a periportal fat accumulation in the left single lobule and an inhomogeneous deposition in the group of lobules. The last example (c) enables the outflow via the neighboring lobules as can be clearly observed in Fig. 12, where the streamlines show the flow direction, which is important for the advective transport of the solutes. The shortest path from the affected lobule to the adjacent upper and lower lobules is decisive and we observe higher external concentrations along this area. In contrast to this, the area between the outflow obstruction and the outflow of the middle lobule shows a reduction of the external concentrations. Here, only diffusion plays a supporting role for the mixture of the solutes in areas with low concentrations. The fat deposition shows how strong the influence of low oxygen concentrations are as these areas have a high-fat fraction, even though there is a reduced concentration of FFAs. In view of these examples, we can conclude that the oxygen gradient is more relevant than the FFA gradient. In case the FFA concentration is insufficient like in situation (b), there is no fat metabolism. Furthermore, we observe a stronger influence of the advective than of the diffusive transport. This indicates a high influence of outflow obstructions on hepatic fat deposition. As expected, a strong correlation between microperfusion and fat metabolism is clearly recognizable and an inhomogeneous fat pattern could be the reason for the disturbed fat metabolism.

## Discussion

Computational models of liver tissue have emerged as tools to quantitatively predict tissue response to stressors and to facilitate clinical translational research (Lerapetritou et al. 2009). However, the fundamental processes of MASLD pathogenesis remain elusive. It is widely accepted, that alterations in hepatic lipid metabolism occur, most likely as a result of a combination of environmental stressors (such as high-fat diet) and genetic predisposition. This lack of knowledge has limited our ability to predict the progression and spatial distribution of MASLD. Currently, only few multiscale whole system models of hepatic fat metabolism are available. Different strategies are pursued taking other factors into account. Maldonado presented an integrated multiscale, whole system model of liver metabolic adaptation to fat and sugar in MASLD. Using this model, he investigated the impact of sugar and fat metabolism on the development of fatty liver disease in cell culture experiments. The proposed kinetic model was incorporated into a hepatocyte specific genome scale metabolic network. As a result, the integrated qualitative model successfully replicated the metabolic response to increased levels of fatty acids, thereby mimicking lipid loading. However, this global model did not take into account hepatic perfusion (Maldonado et al. 2018). Ashworth et al. focused on the modeling of hepatic glucose and fat metabolism taking the zonated expression of metabolic enzymes along the hepatic sinusoid and the sinusoidal blood flow into consideration. In contrast to conventional two-compartment models (blood/hepatocyte) that treat the hepatocyte as the repeating unit of the liver, they considered the porto-central axis of the sinusoid as the repeating unit. They further subdivided the hepatocytes along the sinusoidal axis into eight subcompartments base on their location. Varying the rate constants along the sinusoidal axis according to changes in the zonation of protein expression mediating FFA uptake or very low density lipoprotein synthesis and release determined the fat distribution within the lobule (Ashworth et al. 2016). In contrast, Berndt and his colleagues generated a true multiscale model of hepatic carbohydrate metabolism. This model incorporated not only the intrahepatic blood flow, but also the transport of metabolites between sinusoids and hepatocytes, alongside the metabolic capability of hepatocytes. In order to model the exchange of metabolites, they also considered the position of the hepatocytes along the sinusoids. In addition, they proposed three compartments consisting of the hepatocyte, the space of Disse and the corresponding part of the sinusoid. Furthermore, they investigated the impact of the regional variability of the three determinants on the regional metabolic capacity using sinusoidal tissue units as the smallest repetitive unit. As a main conclusion, they emphasize that regional variability of hepatic blood flow is higher than the corresponding regional variability of the metabolic output (Berndt et al. 2018). Subsequently, the same group expanded their model to encompass fat metabolism. Here, they studied how intrahepatic functional heterogeneity may influence the cascading progression of MASLD. This was achieved by coupling and integrating three modules, a hemodynamic module, a metabolic module and a damage repair module. Within the metabolic module, the liver was likewise partitioned into compact liver units, each composed of two compartments, differentiated based on their positions along the sinusoidal axis. Furthermore, they integrated heterogeneity using areas of different metabolic capacity, as reflected by differences in perfusion distribution. Heterogeneity itself turned out to be a risk factor since metabolic underperformances must be compensated by metabolic overperformance in another area, rendering that area vulnerable to further damage (Holzhütter and Berndt 2021).

The homogenized continuum biomechanical model presented here is a further modification of the computational models reported recently. Our model features four key specifications: (i) It maintains thermodynamic consistency, (ii) it contains three phases (tissue, fat, blood), (iii) it considers five essential substances (glycogen, glucose, lactate, FFA, oxygen), and (iv) it takes two scales (lobule, cell) into consideration. One key difference from the aforementioned models lies in the incorporation of the oxygen gradient. The novelty of this approach is given by for the mutual coupling between spatial and time-dependent liver perfusion, metabolic pathways with a focus on lipid metabolism, and fat accumulation. It is the first time- and spatially resolved homogenized model to account for fatty acid metabolism and resulting fat accumulation in liver lobules. Consequently, the model enables the prediction of fat development in the liver lobule, depending on perfusion, oxygen and plasma FFA content, oxidative processes, and the synthesis of triglycerides. The application of a bi-scale approach enables to integrate scale bridging processes.

Using this thermodynamically consistent multiphase, multicomponent model allowed to simulate the fat metabolism based on the geometry of liver lobules coupled to the physiologically applied microperfusion via the inflow and outflow.

One huge advantage of the simulation is the easy variation of different boundary conditions. Since many aspects of the microperfusion in the liver are still under discussion, we compared three different assumptions for the boundary conditions.

However, the model also has limitations. In order to keep the computing time of the simulations to a minimum and thus enable a broad range of evaluations, a two-dimensional geometry was initially selected, which can, however, easily be extended to include the third dimension. In addition, the model was first extended to include only one fat phase, which represents fat growth. In order to integrate further processes, this model could be extended in a further step by additional phases, such as lymph or a fibrosis. Coupling with broader metabolic processes, such as glucose metabolism, could also provide more detailed information. A more patient-specific simulation is planned by including real lobule geometries by segmentation from histological sections.

In conclusion, we observed that the pressure gradient has a decisive impact on the advective transport of the solutes, which is stronger than the diffusion of the molecules. The local plasma concentrations of metabolites did control the intracellular fat metabolism. A high plasma concentration of FFAs resulted in an increased intracellular fat accumulation; whereas, a high oxygen concentration reduced fat deposition, with oxygen concentration being of greater importance than the concentration of FFA.

The results from this work contribute to a better understanding of the relationship between liver perfusion and function and the influence of MASLD on both parameters. The next step is to validate these mathematical models in experiments and under clinical conditions, initially in healthy subjects. The long-term goal is to apply the models to pathological conditions such as MASLD in order to detect the disease in an early state or even to prevent its onset. Furthermore, the mathematical models could be used in the future to provide a tool for the treatment to better plan oncological operations on the liver or to involve the patient more in the treatment decision in the context of a shared decision process.

Last but not least, a clinical decision support tool can be developed in the future on the basis of computational models, which can help the clinician to make more informed decisions, e.g., in the context of an oncological treatment. Especially patients already suffering from MASLD tolerate, e.g., chemotherapy worse than healthy individuals and suffer more often complications in the course of liver surgery. Here, the benefits and risks must be strictly weighed against each other. However, to achieve this goal, the models still need to be extended in terms of clinical usability. For example, uncertainty analysis can be used to determine the reliability of the results; while, model order reduction methods can be used to reduce computation time.

## Appendix

### Description for the Jacobian $\mathrm J^{\mathrm T}$

Evaluating the balance of mass for a mixture description results in a description for the Jacobian for the fat phase $\textrm{J}^{\textrm{T}}$ of the fat-tissue

36 $$\begin{aligned} \frac{\rho _{\textrm{0}}^{\textrm{T}}}{\rho ^{\textrm{T}}}\,\textrm{exp}(\displaystyle {\int \limits \,\frac{\hat{\rho }^{\textrm{T}}}{\rho ^{\textrm{T}}}\textrm{dt}})\,=\,\textrm{J}^{\textrm{T}}. \end{aligned}$$

With equation (36), the division of the deformation into an elastic $\textrm{J}^{\textrm{T}}_{\textrm{e}}$ and growth $\textrm{J}^{\textrm{T}}_{\textrm{g}}$ part leads to

37 $$\begin{aligned} \textrm{J}^{\textrm{T}}\,=\,\textrm{J}^{\textrm{T}}_{\textrm{e}}\,\textrm{J}^{\textrm{T}}_{\textrm{g}}, \hspace{0.5cm} \textrm{J}^{\textrm{T}}_{\textrm{e}}=\displaystyle \frac{\rho _{\textrm{0}}^{\textrm{T}}}{\rho ^{\textrm{T}}}, \hspace{0.5cm} \textrm{J}^{\textrm{T}}_{\textrm{g}}=\textrm{exp}(\displaystyle {\int \limits \,\frac{\hat{\rho }^{\textrm{T}}}{\rho ^{\textrm{T}}}\textrm{dt}}). \end{aligned}$$

### Evaluation of the entropy inequality

Adding the assumptions from the Helmholtz free energy $\psi ^{{\varvec{\alpha }}}$, a potential which ensures that the released entropy is as large as possible and never decreases in an isolated system, leads to the basic equation for a thermodynamically consistent evaluation, the so-called Clausius–Duhem inequality. Furthermore, the entropy inequality is increased by the derivation of the saturation condition multiplied by the Lagrange parameter $\lambda$. Applying the eTPM approach to these requirements results in the extended entropy inequality for the mixture of phases and microscopic components with

38 $$\begin{aligned} \begin{array}{cll} &{}-&{}\rho ^{\textrm{S}}\, (\psi ^{\textrm{S}})'_{\textrm{S}}\,-\rho ^{\textrm{T}}\,(\psi ^{\textrm{T}})^{\prime }_{\textrm{S}}\,-\rho ^{\textrm{F}}\,(\psi ^{\textrm{F}})'_{\textrm{F}}\, \\ &{}-&{}\rho ^{\textrm{S}\beta }\,(\psi ^{\textrm{S}\beta })^{\prime }_{\mathrm{S\beta }}\,-\,\rho ^{\textrm{F}\beta }\,(\psi ^{\textrm{F}\beta })^{\prime }_{\mathrm{F\beta }}\, \\ {} &{}-&{}\,\hat{\rho }^{\textrm{T}}\,(\,\psi ^{\textrm{T}}\,-\,\frac{\textrm{1}}{\textrm{2}}\,\textbf{x}^{\prime }_{\textrm{S}}\,\cdot \,\textbf{x}^{\prime }_{\textrm{S}})\,\, \\ &{}-&{}\,\hat{\rho }^{\textrm{S}\beta }(\,\psi ^{\textrm{S}\beta }\,-\,\frac{\textrm{1}}{\textrm{2}}\,\textbf{x}^{\prime }_{\textrm{S}}\,\cdot \,\textbf{x}^{\prime }_{\textrm{S}}) \\ {} &{}-&{}\,\hat{\rho }^{\textrm{F}\beta }(\,\psi ^{\textrm{F}\beta }\,-\,\frac{\textrm{1}}{\textrm{2}}\,\textbf{x}^{\prime }_{\textrm{F}\beta }\,\cdot \,\textbf{x}^{\prime }_{\textrm{F}\beta })\, \\ &{}+&{}\,\textbf{T}^{\textrm{S}}\cdot \textbf{D}_{\textrm{S}}\,+\,\textbf{T}^{\textrm{T}}\cdot \textbf{D}_{\textrm{S}}\,+\,\textbf{T}^{\textrm{F}}\cdot \textbf{D}_{\textrm{F}}\, \\ {} &{}+&{} \,\textbf{T}^{\textrm{S}\beta }\cdot \textbf{D}_{\textrm{S}}\,+\,\textbf{T}^{\textrm{F}\beta }\cdot \textbf{D}_{\textrm{F}\beta }\\ {} &{}-&{} \,{\hat{{\textbf { p}}}}^{\textrm{S}}\,\cdot \,\textbf{x}^{\prime }_{\textrm{S}}\,-\,{\hat{{\textbf { p}}}}^{\textrm{T}}\,\cdot \,\textbf{x}^{\prime }_{\textrm{S}}\,-\,{\hat{{\textbf {p}}}}^{\textrm{F}}\,\cdot \,\textbf{x}^{\prime }_{\textrm{F}}\, \\ &{}-&{}\,{\hat{{\textbf { p}}}}^{\textrm{S}\beta }\,\cdot \,\textbf{x}^{\prime }_{\textrm{S}}\,-\,{\hat{{\textbf { p}}}}^{\textrm{F}\beta }\,\cdot \,\textbf{x}^{\prime }_{\textrm{F}\beta }\, \\ {} &{}-&{} \,\lambda \,(-\textbf{D}_{\textrm{S}}\,\cdot \,\textbf{I}\,\textrm{n}^{\textrm{T}}\,+\,\frac{\hat{\rho }^{\textrm{T}}}{\rho ^{\textrm{T}}_{\textrm{R}}} \, - \, \textbf{D}_{\textrm{S}}\,\cdot \,\textbf{I}\,\textrm{n}^{\textrm{S}}\, \, \\ &{}-&{} \textbf{D}_{\textrm{F}}\,\cdot \,\textbf{I}\,\textrm{n}^{\textrm{F}}\,-\,\textrm{grad}\,{\textrm{n}^{\textrm{F}}}\,\cdot \,\textbf{w}_{\textrm{FS}}) \ge \textrm{0}. \end{array} \end{aligned}$$

In order to maintain a consistent approach, the entropy inequality must be fulfilled, which we ensure by applying process variables analogous to Truesdell (1984). The material behavior depends on an independent set of process variables $\mathcal {P}$ as well as on the derivation of the process variables $\mathcal {A}$:

39 $$\begin{aligned} \begin{array}{lcl} \mathcal {P}\,&{}:=&{}\,[\textbf{C}_{\textrm{S}},\,\textrm{n}^{\textrm{S}},\,\textrm{n}^{\textrm{F}},\,\textrm{n}^{\textrm{T}},\,\textbf{w}_{\textrm{F}\beta \textrm{S}},\,\textbf{w}_{\textrm{FS}},\,\textrm{c}^\mathrm{S\beta },\,\textrm{c}^\mathrm{F\beta },\,\\ &{}&{} \,\textrm{c}^\mathrm{T\beta },\,\textrm{grad}\,\textrm{c}^\mathrm{S\beta },\,\textrm{grad}\,\textrm{c}^\mathrm{F\beta },\,\textrm{grad}\,\textrm{c}^\mathrm{T\beta },\,\\ &{}&{} \textrm{grad}\,{\textrm{n}^{\textrm{F}}}] \\ \mathcal {A}\,&{}:=&{}\,[\textbf{D}_{\textrm{S}},\,\textbf{D}_{\textrm{F}},\,\textbf{D}_{\textrm{F}\beta },\,(\textrm{n}^{\textrm{F}})^{\prime }_{\textrm{F}},\,(\textrm{n}^{\textrm{T}})^{\prime }_{\textrm{S}},\,(\textrm{c}^{\textrm{S}\beta })^{\prime }_{\textrm{S}\beta },\, \\ &{}&{} (\textrm{c}^{\textrm{F}\beta })^{\prime }_{\textrm{F}\beta }]. \end{array} \end{aligned}$$

We assume the Helmholtz free energies $\psi ^{\alpha }$ of the phases and microscopic components $\psi ^{\alpha \beta }$ depending on the process variables as follows:

40 $$\begin{aligned} \begin{aligned}&\psi ^{\textrm{S}}\,=\,\psi ^{\textrm{S}}(\textbf{C}_{\textrm{S}}),\,\psi ^{\textrm{T}}\,=\,\psi ^{\textrm{T}}(\textbf{C}_{\textrm{S}}),\,\psi ^{\textrm{F}}\,=\,\psi ^{\textrm{F}}(-),\, \\&\psi ^{\mathrm{S\beta }}_{\textrm{S}}\,=\,\psi ^{\mathrm{S\beta }}_{\textrm{S}}(\textrm{c}^\mathrm{S\beta }),\,\psi ^{\mathrm{F\beta }}_{\textrm{F}}\,=\,\psi ^{\mathrm{F\beta }}_{\textrm{F}}(\textrm{c}^\mathrm{F\beta }). \end{aligned} \end{aligned}$$

Therein, $\psi ^{\mathrm{S\beta }}_{\textrm{S}}$ and $\psi ^{\mathrm{F\beta }}_{\textrm{F}}$ describe the energies depending on the realistic volume of the microscopic components. Resulting in the relation of partial energy $\psi ^{\alpha \beta }$ and the volume-specific energy $\psi ^{\alpha \beta }_{\alpha }$ with:

41 $$\begin{aligned} \psi ^{\mathrm{S\beta }}_{\textrm{S}}\,=\,\rho ^{\textrm{S}\beta }_{\textrm{R}}\,\psi ^{\mathrm{S\beta }}, \hspace{2cm} \psi ^{\mathrm{F\beta }}_{\textrm{F}}\,=\,\rho ^{\textrm{F}\beta }_{\textrm{R}}\,\psi ^{\mathrm{F\beta }}. \end{aligned}$$

The temporal changes of the Helmholtz free energies depend on the process variables as follows:

42 $$\begin{aligned} \begin{array}{lcl} \rho ^{\textrm{S}}\,(\psi ^{\textrm{S}})'_{\textrm{S}}\,&{}=&{}\,\textrm{2}\,\rho ^{\textrm{S}}\,\textbf{F}_{\textrm{S}}\,\displaystyle {\frac{\partial \psi ^{\textrm{S}}}{\partial \textbf{C}_{\textrm{S}}}}\,\textbf{F}_{\textrm{S}}^{\textrm{T}}\,\cdot \,\textbf{D}_{\textrm{S}}, \\ \rho ^{\textrm{T}}\,(\psi ^{\textrm{T}})^{\prime }_{\textrm{S}}\,&{}=&{}\,\textrm{2}\,\rho ^{\textrm{T}}\,\textbf{F}_{\textrm{S}}\,\displaystyle {\frac{\partial \psi ^{\textrm{T}}}{\partial \textbf{C}_{\textrm{S}}}}\,\textbf{F}_{\textrm{S}}^{\textrm{T}}\,\cdot \,\textbf{D}_{\textrm{S}}, \\ \rho ^{\textrm{F}}\,(\psi ^{\textrm{F}})'_{\textrm{F}}\,&{}=&{}\,\textrm{0}. \\ \end{array} \end{aligned}$$

As the energy functions of the microscopic components $\psi ^{\mathrm{S\beta }}_{\textrm{S}}$ and $\psi ^{\mathrm{F\beta }}_{\textrm{F}}$ depend on the realistic volume (41), we have to apply the chain rule for the evaluation.

43 $$\begin{aligned} \begin{array}{lcl} \rho ^{\textrm{F}\beta }\,(\psi ^{\textrm{F}\beta })^{\prime }_{\mathrm{\beta }}\,&{}=&{}\,\textrm{n}^{\textrm{F}}\,{(\psi ^{\textrm{F}\beta }_{\textrm{F}})}^{\prime }_{\mathrm{\beta }}\,-\,\textrm{n}^{\textrm{F}}\,\psi ^{\textrm{F}\beta }\,({\rho ^{\textrm{F}\beta }_{\textrm{R}}})^{\prime }_{\mathrm{\beta }} \\ \rho ^{\textrm{S}\beta }\,(\psi ^{\textrm{S}\beta })^{\prime }_{\mathrm{\beta }}\,&{}=&{}\,\textrm{n}^{\textrm{S}}\,{(\psi ^{\textrm{S}\beta }_{\textrm{S}})}^{\prime }_{\mathrm{\beta }}\,-\,\textrm{n}^{\textrm{S}}\,\psi ^{\textrm{S}\beta }\,({\rho ^{\textrm{S}\beta }_{\textrm{R}}})^{\prime }_{\mathrm{\beta }}. \end{array} \end{aligned}$$

We need further transformations for the derivation of the volume-specific energy, which can be reformulated into

44 $$\begin{aligned} \begin{array}{lcl} (\psi ^{\textrm{F}\beta }_{\textrm{F}})^{\prime }_{\mathrm{\beta }}&{}=&{}\,(\psi ^{\textrm{F}\beta }_{\textrm{F}})^{\prime }_{\textrm{F}}\,-\,\textrm{grad}\,\psi ^{\textrm{F}\beta }_{\textrm{F}}\cdot \textbf{w}_{\textrm{FS}}\,\\ &{}+&{}\,\textrm{grad}\,\psi ^{\textrm{F}\beta }_{\textrm{F}}\cdot \textbf{w}_{\textrm{F}\beta \textrm{S}}\\ (\psi ^{\textrm{S}\beta }_{\textrm{S}})^{\prime }_{\mathrm{\beta }}&{}=&{}\,(\psi ^{\textrm{S}\beta }_{\textrm{S}})^{\prime }_{\textrm{S}}. \end{array} \end{aligned}$$

From the balance equations of the microscopic components in the fluid (13_(4,5)_), we gain the relations:

45 $$\begin{aligned} 
\begin{array}{lcl} \textrm{n}^{\textrm{F}}\,({\rho ^{\textrm{F}\beta }_{\textrm{R}}})^{\prime }_{\mathrm{\beta }}\,&{}=&{}\,-\,(\textrm{n}^{\textrm{F}})^{\prime }_{\mathrm{\beta }}\,\rho ^{\textrm{F}\beta }_{\textrm{R}}\,-\,\textrm{n}^{\textrm{F}}\,\rho ^{\textrm{F}\beta }_{\textrm{R}}\,\textrm{div}\,\textbf{x}^{\prime }_{\beta }\,+\,\hat{\rho }^{\textrm{F}\beta }\\ \textrm{n}^{\textrm{S}}\,({\rho ^{\textrm{S}\beta }_{\textrm{R}}})^{\prime }_{\mathrm{\beta }}\,&{}=&{}\,\hat{\rho }^{\textrm{S}\beta }. \end{array} \end{aligned}$$

Herein, we solve $(\textrm{n}^{\textrm{F}})^{\prime }_{\mathrm{\beta }}$ to

46 $$\begin{aligned} (\textrm{n}^{\textrm{F}})^{\prime }_{\mathrm{\beta }}\,=\,-\textbf{D}_{\textrm{F}}\,\cdot \,\textbf{I}\,-\,\textrm{grad}\,{\textrm{n}^{\textrm{F}}}\,\textbf{w}_{\textrm{FS}}\,+\,\textrm{grad}\,{\textrm{n}^{\textrm{F}}}\,\textbf{w}_{\textrm{F}\beta \textrm{S}}. \end{aligned}$$

The volume-specific energy of the fluid $\psi ^{\textrm{F}\beta }_{\textrm{F}}$ depends on the process variable of the external microscopic concentration $\textrm{c}^\mathrm{F\beta }$,

47 $$\begin{aligned} (\psi ^{\textrm{F}\beta }_{\textrm{F}}(\textrm{c}^\mathrm{F\beta }))^{\prime }_{\textrm{F}}= & {} \,\frac{\partial \psi ^{\textrm{F}\beta }_{\textrm{F}}}{\partial \textrm{c}^\mathrm{F\beta }}\,(\textrm{c}^{\textrm{F}\beta })^{\prime }_{\textrm{F}\beta }\,-\,\textrm{grad}\,\psi ^{\textrm{F}\beta }_{\textrm{F}}\,\cdot \,\textbf{w}_{\textrm{F}\beta \textrm{S}}\,\nonumber \\{} & {} +\,\textrm{grad}\,\psi ^{\textrm{F}\beta }_{\textrm{F}}\,\cdot \,\textbf{w}_{\textrm{FS}}\end{aligned}$$

48 $$\begin{aligned} (\textrm{c}^{\textrm{F}\beta })^{\prime }_{\textrm{F}\beta }\,= & {} \,\textrm{c}^\mathrm{F\beta }\,\textbf{D}_{\textrm{F}}\,\cdot \,\textbf{I}\,-\,\frac{1}{\textrm{n}^{\textrm{F}}}\,\textrm{grad}\,{\textrm{n}^{\textrm{F}}}\,\textbf{w}_{\textrm{F}\beta \textrm{S}}\,\textrm{c}^\mathrm{F\beta }\,\nonumber \\{} & {} +\,\frac{1}{\textrm{n}^{\textrm{F}}}\,\textrm{grad}\,{\textrm{n}^{\textrm{F}}}\,\textbf{w}_{\textrm{FS}}\,\textrm{c}^\mathrm{F\beta }\,-\,\textrm{c}^\mathrm{F\beta }\,\textbf{D}_{\textrm{F}\beta }\,\cdot \,\textbf{I}\,\nonumber \\{} & {} -\,\frac{\hat{\rho }^{\textrm{F}\beta }}{\textrm{n}^{\textrm{F}}\,\textrm{M}^{\textrm{F}\beta }_\textrm{mol}}. \end{aligned}$$

Furthermore, the volume-specific energy of the solid $\psi ^{\textrm{S}\beta }_{\textrm{S}}$ depends on the process variable of the internal microscopic concentration $\textrm{c}^\mathrm{S\beta }$,

49 $$\begin{aligned} \begin{aligned} (\psi ^{\textrm{S}\beta }_{\textrm{S}}(\textrm{c}^\mathrm{S\beta }))^{\prime }_{\textrm{S}}&=\,\frac{\partial \psi ^{\textrm{S}\beta }_{\textrm{S}}}{\partial \textrm{c}^\mathrm{S\beta }}\,(\textrm{c}^{\textrm{S}\beta })^{\prime }_{\textrm{S}\beta }, \\ (\textrm{c}^{\textrm{S}\beta })^{\prime }_{\textrm{S}\beta }\,&=\,\frac{\hat{\rho }^{\textrm{S}\beta }}{\textrm{n}^{\textrm{S}}\,\textrm{M}^{\textrm{S}\beta }_\textrm{mol}}. \end{aligned} \end{aligned}$$

In the end, we get the equations for the temporal changes of the Helmholtz free energies

50 $$\begin{aligned} \begin{array}{cll} \rho ^{\textrm{F}\beta }\,(\psi ^{\textrm{F}\beta })^{\prime }_{\mathrm{\beta }}\,&{}=&{}\,\displaystyle \frac{\partial \psi ^{\textrm{F}\beta }_{\textrm{F}}}{\partial \textrm{c}^\mathrm{F\beta }}\,(\textrm{n}^{\textrm{F}}\,\textrm{c}^\mathrm{F\beta }\,\textbf{D}_{\textrm{F}}\,\cdot \,\textbf{I}\,\\ {} &{}-&{}\,\textrm{grad}\,{\textrm{n}^{\textrm{F}}}\,\textbf{w}_{\textrm{F}\beta \textrm{S}}\,\textrm{c}^\mathrm{F\beta }\, \\ {} &{}+&{}\,\textrm{grad}\,{\textrm{n}^{\textrm{F}}}\,\textbf{w}_{\textrm{FS}}\,\textrm{c}^\mathrm{F\beta }\, \\ &{}-&{}\,\textrm{n}^{\textrm{F}}\,\textrm{c}^\mathrm{F\beta }\,\textbf{D}_{\textrm{F}\beta }\,\cdot \,\textbf{I}\,-\displaystyle \frac{\hat{\rho }^{\textrm{F}\beta }}{\textrm{M}^{\textrm{F}\beta }_\textrm{mol}}) \\ &{}=&{}\psi ^{\textrm{F}\beta }_{\textrm{F}}(\textrm{n}^{\textrm{F}}\,\textbf{D}_{\textrm{F}}\,\cdot \,\textbf{I}\,+\,\textrm{grad}\,{\textrm{n}^{\textrm{F}}}\,\textbf{w}_{\textrm{FS}}\,\\ &{}-&{}\,\textrm{grad}\,{\textrm{n}^{\textrm{F}}}\,\textbf{w}_{\textrm{F}\beta \textrm{S}}\,-\,\textrm{n}^{\textrm{F}}\,\textbf{D}_{\beta }\,\cdot \,\textbf{I}\,\\ {} &{}-&{}\displaystyle \frac{\hat{\rho }^{\textrm{F}\beta }}{\rho ^{\textrm{F}\beta }_{\textrm{R}}}) \\ \rho ^{\textrm{S}\beta }\,(\psi ^{\textrm{S}\beta })^{\prime }_{\mathrm{\beta }}\,&{}=&{}\displaystyle \frac{\partial \psi ^{\textrm{S}\beta }_{\textrm{S}}}{\partial \textrm{c}^\mathrm{S\beta }}\,\frac{\hat{\rho }^{\textrm{S}\beta }}{\,\textrm{M}^{\textrm{S}\beta }_\textrm{mol}}\,-\,\psi ^{\textrm{S}\beta }_{\textrm{S}}\,\frac{\hat{\rho }^{\textrm{S}\beta }}{\rho ^{\textrm{S}\beta }_{\textrm{R}}}. \end{array} \end{aligned}$$

The elastic part of the entropy inequality can be reformulated and sorted by the process variables

51 $$\begin{aligned} \begin{array}{lcll} \textbf{D}_{\textrm{S}}&{}\cdot &{}\,(\textbf{T}^{\textrm{S}}\,+\,\textbf{T}^{\textrm{T}}\,+\,\textbf{T}^{\textrm{S}\beta }\,+\,\lambda \,\textbf{I}\,(\,\textrm{n}^{\textrm{S}}\,+\,\textrm{n}^{\textrm{T}})\,\\ &{}-&{}\textrm{2}\,\rho ^{\textrm{S}}\,\textbf{F}_{\textrm{S}}\,\displaystyle {\frac{\partial \psi ^{\textrm{S}}}{\partial \textbf{C}_{\textrm{S}}}}\,\textbf{F}_{\textrm{S}}^{\textrm{T}}\,-\,2\,\rho ^{\textrm{T}}\,\textbf{F}_{\textrm{S}}\,\displaystyle {\frac{\partial \psi ^{\textrm{T}}}{\partial \textbf{C}_{\textrm{S}}}}\,\textbf{F}_{\textrm{S}}^{\textrm{T}}) &{} \\ \textbf{D}_{\textrm{F}}&{}\cdot &{}\,(\textbf{T}^{\textrm{F}}\,+\displaystyle \displaystyle \frac{\partial \psi ^{\textrm{F}\beta }_{\textrm{F}}}{\partial \textrm{c}^\mathrm{F\beta }}\,\textrm{n}^{\textrm{F}}\,\textrm{c}^\mathrm{F\beta }\,\textbf{I}\,-\,\psi ^{\textrm{F}\beta }_{\textrm{F}}\,\textrm{n}^{\textrm{F}}\,\textbf{I}\,\\ &{}&{}+\,\lambda \,\textbf{I}\,\,\textrm{n}^{\textrm{F}}) &{} \\ \textbf{D}_{\textrm{F}\beta }&{}\cdot &{}\,(\textbf{T}^{\textrm{F}\beta }\,-\displaystyle \frac{\partial \psi ^{\textrm{F}\beta }_{\textrm{F}}}{\partial \textrm{c}^\mathrm{F\beta }}\,\textrm{n}^{\textrm{F}}\,\textrm{c}^\mathrm{F\beta }\,\textbf{I}\,+\,\psi ^{\textrm{F}\beta }_{\textrm{F}}\,\textrm{n}^{\textrm{F}}\,\textbf{I}) = 0.&{} \end{array} \end{aligned}$$

From the evaluation of the elastic part of the entropy inequality we gain the following restrictions for the stresses:

52 $$\begin{aligned} \begin{array}{lcll} \textbf{T}^{\textrm{S}}+\textbf{T}^{\textrm{T}}+\textbf{T}^{\textrm{S}\beta }\, &{}=&{}\,\textrm{2}\,\rho ^{\textrm{S}}\,\textbf{F}_{\textrm{S}}\,\displaystyle {\frac{\partial \psi ^{\textrm{S}}}{\partial \textbf{C}_{\textrm{S}}}}\,\textbf{F}_{\textrm{S}}^{\textrm{T}}\, \\ &{}\quad+&{}\,\textrm{2}\,\rho ^{\textrm{T}}\,\textbf{F}_{\textrm{S}}\,\displaystyle {\frac{\partial \psi ^{\textrm{T}}}{\partial \textbf{C}_{\textrm{S}}}}\,\textbf{F}_{\textrm{S}}^{\textrm{T}}\, \\ &{}-&{}\,\lambda \,\textbf{I}\,(\,\textrm{n}^{\textrm{S}}\,+\,\textrm{n}^{\textrm{T}}) \\ \textbf{T}^{\textrm{F}}&{}=&{}\,\quad-\displaystyle \frac{\partial \psi ^{\textrm{F}\beta }_{\textrm{F}}}{\partial \textrm{c}^\mathrm{F\beta }}\,\textrm{n}^{\textrm{F}}\,\textrm{c}^\mathrm{F\beta }\,\textbf{I}\,+\,\psi ^{\textrm{F}\beta }_{\textrm{F}}\,\textrm{n}^{\textrm{F}}\,\textbf{I}\,\\ {} &{}-&{}\,\lambda \,\textbf{I}\,\,\textrm{n}^{\textrm{F}}\\ \textbf{T}^{\textrm{F}\beta }&{}\quad=&{}\,\displaystyle \frac{\partial \psi ^{\textrm{F}\beta }_{\textrm{F}}}{\partial \textrm{c}^\mathrm{F\beta }}\,\textrm{n}^{\textrm{F}}\,\textrm{c}^\mathrm{F\beta }\,\textbf{I}\,-\,\psi ^{\textrm{F}\beta }_{\textrm{F}}\,\textrm{n}^{\textrm{F}}\,\textbf{I}\end{array} \end{aligned}$$

Subsequently, the dissipative parts of the entropy inequality result in

53 $$\begin{aligned} \begin{array}{lll} -\textbf{w}_{\textrm{FS}}&{}\cdot \,({\hat{{\textbf {p}}}}^{\textrm{F}}\,-\,\lambda \,\textrm{grad}\,{\textrm{n}^{\textrm{F}}}\,-\,\displaystyle \frac{\partial \psi ^{\textrm{F}\beta }_{\textrm{F}}}{\partial \textrm{c}^\mathrm{F\beta }}\,\textrm{grad}\,{\textrm{n}^{\textrm{F}}}\,\textrm{c}^\mathrm{F\beta }\, \\ {} &{}+\,\psi ^{\textrm{F}\beta }_{\textrm{F}}\,\textrm{grad}\,{\textrm{n}^{\textrm{F}}}) \\ -\textbf{w}_{\textrm{F}\beta \textrm{S}}&{}\cdot \,({\hat{{\textbf { p}}}}^{\textrm{F}\beta }\,+\,\displaystyle \frac{\partial \psi ^{\textrm{F}\beta }_{\textrm{F}}}{\partial \textrm{c}^\mathrm{F\beta }}\,\textrm{grad}\,{\textrm{n}^{\textrm{F}}}\,\textrm{c}^\mathrm{F\beta }\,-\,\psi ^{\textrm{F}\beta }_{\textrm{F}}\,\textrm{grad}\,{\textrm{n}^{\textrm{F}}}) \\ -\hat{\rho }^{\textrm{T}}&\quad{}\cdot \,(\psi ^{\textrm{T}}\,-\,\frac{\textrm{1}}{\textrm{2}}\,\textbf{x}^{\prime }_{\textrm{S}}\,\cdot \,\textbf{x}^{\prime }_{\textrm{S}}\,+\,\frac{\lambda }{\rho 
^{\textrm{T}}_{\textrm{R}}}) \\ -\hat{\rho }^{\textrm{S}\beta }&\quad{}\cdot \,(\psi ^{\textrm{S}\beta }\,-\,\frac{\textrm{1}}{\textrm{2}}\,\textbf{x}^{\prime }_{\textrm{S}}\,\cdot \,\textbf{x}^{\prime }_{\textrm{S}}\,+\,\displaystyle \frac{\partial \psi ^{\textrm{S}\beta }_{\textrm{S}}}{\partial \textrm{c}^\mathrm{S\beta }}\,\displaystyle \frac{\textrm{1}}{\,\textrm{M}^{\textrm{S}\beta }_\textrm{mol}}\, \\ {} &\quad{}+\,\psi ^{\textrm{S}\beta }_{\textrm{S}}\,\frac{\textrm{1}}{\rho ^{\textrm{S}\beta }_{\textrm{R}}}) \\ -\hat{\rho }^{\textrm{F}\beta }&\quad{}\cdot \,(\psi ^{\textrm{F}\beta }\,-\,\displaystyle \frac{\textrm{1}}{\textrm{2}}\,\textbf{x}^{\prime }_{\textrm{F}\beta }\,\cdot \,\textbf{x}^{\prime }_{\textrm{F}\beta }\,+\,\displaystyle \frac{\partial \psi ^{\textrm{F}\beta }_{\textrm{F}}}{\partial \textrm{c}^\mathrm{F\beta }}\,\frac{\textrm{1}}{\,\textrm{M}^{\textrm{F}\beta }_\textrm{mol}}\,\\ {} &\quad{}+\,\psi ^{\textrm{F}\beta }_{\textrm{F}}\,\displaystyle \frac{\textrm{1}}{\rho ^{\textrm{F}\beta }_{\textrm{R}}}) \\ &{}\ge 0 \end{array} \end{aligned}$$

and yield the restrictions

54 $$\begin{aligned} \begin{array}{lcll} \bar{\psi }^{\textrm{F}\beta }\,&\quad{}=&{}\,\psi ^{\textrm{F}\beta }\,-\,\displaystyle \frac{\textrm{1}}{\textrm{2}}\,\textbf{x}^{\prime }_{\textrm{F}\beta }\,\cdot \,\textbf{x}^{\prime }_{\textrm{F}\beta }\,+\,\displaystyle \frac{\partial \psi ^{\textrm{F}\beta }_{\textrm{F}}}{\partial \textrm{c}^\mathrm{F\beta }}\,\displaystyle \frac{\textrm{1}}{\,\textrm{M}^{\textrm{F}\beta }_\textrm{mol}}\,\\ {} &{}\quad+&{}\,\psi ^{\textrm{F}\beta }_{\textrm{F}}\,\frac{\textrm{1}}{\rho ^{\textrm{F}\beta }_{\textrm{R}}} &\quad{} \\ \bar{\psi }^{\textrm{S}\beta }\,&{}=&{}\,\psi ^{\textrm{S}\beta }\,-\,\displaystyle \frac{\textrm{1}}{\textrm{2}}\,\textbf{x}^{\prime }_{\textrm{S}}\,\cdot \,\textbf{x}^{\prime }_{\textrm{S}}\,+\,\displaystyle \frac{\partial \psi ^{\textrm{S}\beta }_{\textrm{S}}}{\partial \textrm{c}^\mathrm{S\beta }}\,\frac{\textrm{1}}{\,\textrm{M}^{\textrm{S}\beta }_\textrm{mol}}\, \\ {} &{}&{}+\,\psi ^{\textrm{S}\beta }_{\textrm{S}}\,\displaystyle \frac{\textrm{1}}{\rho ^{\textrm{S}\beta }_{\textrm{R}}} &{} \\ \bar{\psi }^{\textrm{T}}\,&\quad{}=&{}\,\psi ^{\textrm{T}}\,-\,\displaystyle \frac{\textrm{1}}{\textrm{2}}\,\textbf{x}^{\prime }_{\textrm{S}}\,\cdot \,\textbf{x}^{\prime }_{\textrm{S}}+\,\displaystyle \frac{\lambda }{\rho ^{\textrm{T}}_{\textrm{R}}} &{} \\ {\hat{{\textbf {p}}}}^{\textrm{F}}\,&{}=&{}\,\lambda \,\textrm{grad}\,{\textrm{n}^{\textrm{F}}}\,+\,\displaystyle \frac{\partial \psi ^{\textrm{F}\beta }_{\textrm{F}}}{\partial \textrm{c}^\mathrm{F\beta }}\,\textrm{grad}\,{\textrm{n}^{\textrm{F}}}\,\textrm{c}^\mathrm{F\beta }\, \\ {} &{}\quad-&{}\,\psi ^{\textrm{F}\beta }_{\textrm{F}}\,\textrm{grad}\,{\textrm{n}^{\textrm{F}}}\, -\,\gamma ^\textrm{F}_{\textbf{w}_{\textrm{FS}}}\,\textbf{w}_{\textrm{FS}}\,+\,\,\gamma ^\textrm{F}_{\textbf{w}_{\textrm{F}\beta \textrm{S}}}\,\textbf{w}_{\textrm{F}\beta \textrm{S}}&{} \\ {\hat{{\textbf { p}}}}^{\textrm{F}\beta }\,&{}=&{}\,\quad-\displaystyle \frac{\partial \psi ^{\textrm{F}\beta }_{\textrm{F}}}{\partial \textrm{c}^\mathrm{F\beta }}\,\textrm{grad}\,{\textrm{n}^{\textrm{F}}}\,\textrm{c}^\mathrm{F\beta }\,+\,\psi ^{\textrm{F}\beta }_{\textrm{F}}\,\textrm{grad}\,{\textrm{n}^{\textrm{F}}}\, \\ &{}-&{}\,\gamma ^\textrm{F}_{\textbf{w}_{\textrm{F}\beta \textrm{S}}}\,\textbf{w}_{\textrm{F}\beta \textrm{S}}\,-\,\,\gamma ^\textrm{F}_{\textbf{w}_{\textrm{FS}}}\,\textbf{w}_{\textrm{FS}}. \end{array} \end{aligned}$$

### Stresses and interaction forces

From the entropy evaluation we get a restriction for the stresses, interaction forces and for the mass-exchange. Together with this, we apply the Helmholtz free energy function for a Neo Hookean solid with

55 $$\begin{aligned} \begin{array}{lcl} \psi ^{\textrm{S}}\,&\quad{}=&{}\,\displaystyle {\frac{\textrm{1}}{\rho ^{\textrm{S}}_{\textrm{0S}}}}\,\left( \lambda ^{\textrm{S}}\,\frac{\textrm{1}}{\textrm{2}}\,(\textrm{ln}\,\textrm{J}_{\textrm{S}})^2\,-\,\mu ^{\textrm{S}}\,\textrm{ln}\,\textrm{J}_{\textrm{S}}\, \displaystyle \right. \\ {} &\quad{}+&{}\,\left. \frac{\textrm{1}}{\textrm{2}}\,\mu ^{\textrm{S}}\,(\textrm{tr}\,\textbf{C}_{\textrm{S}}\,-\,\textrm{3})\displaystyle \right) ,\\ \psi ^{\textrm{T}}\, &{}=&{}\,\displaystyle {\frac{\textrm{1}}{\rho ^{\textrm{T}}_{\textrm{0S}}}}\,\left( \lambda ^{\textrm{T}}\,\frac{\textrm{1}}{\textrm{2}}\,(\textrm{ln}\,\textrm{J}_{\textrm{S}})^2\,-\,\mu ^{\textrm{T}}\,\textrm{ln}\,\textrm{J}_{\textrm{S}}\, \right. \\ {} &\quad{}+&{} \left. \displaystyle \,\frac{\textrm{1}}{\textrm{2}}\,\mu ^{\textrm{T}}\,(\textrm{tr}\,\textbf{C}_{\textrm{S}}\,-\,\textrm{3})\right) , \end{array} \end{aligned}$$

where $\lambda ^{\textrm{S}}$, $\lambda ^{\textrm{T}}$ and $\mu ^{\textrm{S}}$, $\mu ^{\textrm{T}}$ are the Lamé constants. The derivation by the process variable $\textbf{C}_{\textrm{S}}$ follows with

56 $$\begin{aligned} \begin{array}{cll} \displaystyle {\frac{\partial \psi ^{\text{S}}}{\partial \textbf{C}_{\text{S}}}}\,&{}=&{}\,\displaystyle {\frac{ {1}}{\rho ^{\text{S}}_{\text{0S}}}}\,\left( \lambda ^{\text{S}}\,\frac{ {1}}{ {2}}\,\textbf{C}_{\text{S}}^{{-1}}\,\text{ln}\,\text{J}_{\text{S}}\, \right. \\&{}&{} \left. -\,\mu ^{\text{S}}\,\frac{ {1}}{ {2}}\,\textbf{C}_{\text{S}}^{{-1}}\,+\,\frac{ {1}}{ {2}}\,\mu ^{\text{S}}\,\textbf{I}\right) \\ \\ \displaystyle {\frac{\partial \psi ^{\text{T}}}{\partial \textbf{C}_{\text{S}}}}\,&{}=&{}\,\displaystyle {\frac{ {1}}{\rho ^{\text{T}}_{\text{0S}}}}\,\left( \lambda ^{\text{T}}\,\frac{ {1}}{ {2}}\,\textbf{C}_{\text{S}}^{\mathrm{-1}}\,\text{ln}\,\text{J}_{\text{S}}\,\right. \\ &{}&{} \left. -\,\mu ^{\text{T}}\,\frac{ {1}}{ {2}}\,\textbf{C}_{\text{S}}^{{-1}}\,+\,\frac{ {1}}{ {2}}\,\mu ^{\text{T}}\,\textbf{I}\right) \,. \end{array} \end{aligned}$$

The Helmholtz free energy function for the microscopic components $\psi ^{\textrm{F}\beta }_{\textrm{F}}$ is applied according to Acartürk (2009), Ehlers (2009) and Wagner (2014). The chemical potential $\mu ^{\textrm{F}\beta }$ is obtained by the derivation of the ansatz with respect to the process variable $\textrm{c}^\mathrm{F\beta }$.

57 $$\begin{aligned} \begin{array}{lcll} \psi ^{\textrm{F}\beta }_{\textrm{F}} &{}=&{}\,\textrm{c}^\mathrm{F\beta }\,\mu ^{\textrm{F}\beta }_{0}\,+\,\textrm{c}^\mathrm{F\beta }\,\textrm{R}\,\theta \,(\ln \,\textrm{c}^\mathrm{F\beta }\,-\,\textrm{1}) \\ \displaystyle \frac{\partial \psi ^{\textrm{F}\beta }_{\textrm{F}}}{\partial \textrm{c}^\mathrm{F\beta }} &{}=&{}\,\mu ^{\textrm{F}\beta }_{0}\,+\,\textrm{R}\,\theta \,\ln \,\textrm{c}^\mathrm{F\beta }\,=\,\mu ^{\textrm{F}\beta }. \end{array} \end{aligned}$$

The chemical potential founded on restrictions from the entropy inequality yields a function of the solute concentrations. This is used for the calculation of the concentrations as

58 $$\begin{aligned} \textrm{c}^{\alpha \beta }\,=\,\displaystyle {\textrm{e}^{ {\displaystyle \frac{\textrm{M}^{\beta }_\textrm{mol}}{\textrm{R}\,\theta }\,(\mu ^{\textrm{F}}\,-\,\mu ^{\alpha \beta }_{\textrm{0}})}}}\,\textrm{c}^{\alpha \beta }_{\textrm{0}}. \end{aligned}$$

The approach includes the general gas constant $\textrm{R}$, the temperature of the mixture $\theta$ and the constant standard potential $\mu ^{\textrm{F}\beta }_{0}$. Analogous to Acartürk (2009), Ehlers (2009), Bowen (1980, 1976) and Wagner (2014), we can distinguish between osmotic pressure $\mathrm{\Pi }$, resulting in the stress of the external concentrations

59 $$\begin{aligned} \mathrm{\Pi }\,=\,\textrm{R}\,\theta \,\sum _{\textrm{F}\beta }\textrm{c}^\mathrm{F\beta }\end{aligned}$$

and hydrostatic pressure $\lambda$, identified by the Lagrange parameter.

The total pressure is calculated by the sum of hydrostatic and osmotic pressure (59) as

60 $$\begin{aligned} {\text {p}}^{\text {R}}\,=\,\text {p}^{\text {FR}}\,+\,\mathrm{\Pi }. \end{aligned}$$

Inserting the approaches for the Helmholtz free energy functions (55, 57_(1)_) and their derivations (56, 57_(2)_) in the evaluation of the entropy inequality results in the following stress equations:

61 $$\begin{aligned} \begin{array}{lcll} \textbf{T}^{\textrm{S}}\,+\,\textbf{T}^{\textrm{T}}\,+\,\textbf{T}^{\textrm{S}\beta }\, &{}=&{}\,\displaystyle {\frac{\rho ^{\textrm{S}}}{\rho ^{\textrm{S}}_{\textrm{0S}}}}\left( \lambda ^{\textrm{S}}\,\textrm{ln}\,\textrm{J}_{\textrm{S}}\,\textbf{I}\,+\,\textrm{2}\,\mu ^{\textrm{S}}\,\textbf{K}_{\textrm{S}}\right) \, \\ {} &{}+&{}\,\quad\displaystyle {\frac{\rho ^{\textrm{T}}}{\rho ^{\textrm{T}}_{\textrm{0S}}}}\left( \lambda ^{\textrm{T}}\,\textrm{ln}\,\textrm{J}_{\textrm{S}}\,\textbf{I}\,+\,\textrm{2}\,\mu ^{\textrm{T}}\,\textbf{K}_{\textrm{S}}\right) \, \\ {} &\quad{}-&{}\,\lambda \,\textbf{I}\,(\,\textrm{n}^{\textrm{S}}\,+\,\textrm{n}^{\textrm{T}}) \\ \textbf{T}^{\textrm{F}}&{}=\quad&{}\,-\textrm{n}^{\textrm{F}}\,(\lambda \,+\mathrm{\Pi })\,\textbf{I}\\ \textbf{T}^{\textrm{F}\beta }&{}=\quad&{}\,\textrm{n}^{\textrm{F}}\,\textrm{c}^\mathrm{F\beta }\textrm{R}\,\theta \,\textbf{I}\,=\,\textrm{n}^{\textrm{F}}\,\mathrm{\Pi }\,\textbf{I}\end{array} \end{aligned}$$

with the Karni-Reiner stress tensor $\textbf{K}_{\textrm{S}}$. The restrictions for the dissipative momentum production follow as

62 $$\begin{aligned} \begin{array}{lcll} {\hat{{\textbf {p}}}}^{\textrm{F}}\,&{}=&{}\,\textrm{grad}\,{\textrm{n}^{\textrm{F}}}\,\lambda \,+\,\mathrm{\Pi }\,\textrm{grad}\,{\textrm{n}^{\textrm{F}}}\,+\,\gamma ^\textrm{F}_{\textbf{w}_{\textrm{F}\beta \textrm{S}}}\,\textbf{w}_{\textrm{F}\beta \textrm{S}}\, \\ {} &{}-&{}\,\gamma ^\textrm{F}_{\textbf{w}_{\textrm{FS}}}\,\textbf{w}_{\textrm{FS}}\\ {\hat{{\textbf {p}}}}^{\textrm{F}\beta }\,&{}=&{}\,-\mathrm{\Pi }\,\textrm{grad}\,{\textrm{n}^{\textrm{F}}}\,-\,\gamma ^\textrm{F}_{\textbf{w}_{\textrm{F}\beta \textrm{S}}}\,\textbf{w}_{\textrm{F}\beta \textrm{S}}\,-\,\gamma ^\textrm{F}_{\textbf{w}_{\textrm{FS}}}\,\textbf{w}_{\textrm{FS}}. \\ \end{array} \end{aligned}$$

The restrictions for the interaction forces enable the draft of a constitutive law for the filter velocity. First, we focus on the flux of the external concentrations $\textbf{w}_{\textrm{F}\beta \textrm{S}}$, that consist of miscible components solved within the fluid. For this, we apply the Cauchy stresses (61) and momentum production (62), to recalculate the balance of momentum for the fluid components to

63 $$\begin{aligned} \begin{array}{lcll} \textrm{n}^{\textrm{F}}\,\textrm{grad}\, \mathrm{\Pi }\,-\,\gamma ^\textrm{F}_{\textbf{w}_{\textrm{F}\beta \textrm{S}}}\,\textbf{w}_{\textrm{F}\beta \textrm{S}}\,-\,\gamma ^\textrm{F}_{\textbf{w}_{\textrm{FS}}}\,\textbf{w}_{\textrm{FS}}\,= & {} \textbf{0}. \end{array} \end{aligned}$$

The seepage velocity of the external compounds is obtained:

64 $$\textbf{w}_{\textrm{F}\beta \textrm{S}}\,=\,\displaystyle \frac{\textrm{1}}{\gamma ^\textrm{F}_{\textbf{w}_{\textrm{F}\beta \textrm{S}}}}\,(\textrm{n}^{\textrm{F}}\,\textrm{grad}\, \mathrm{\Pi }\,-\,\gamma ^\textrm{F}_{\textbf{w}_{\textrm{FS}}}\,\textbf{w}_{\textrm{FS}}). $$

Note that in equation (64) the division into two parts is obvious, as the motion of the external concentration is influenced on one hand by advection of the carrier phase and on the other hand by the diffusion of the concentrations. We can identify the diffusive motion driven by the osmotic pressure $\textrm{grad}\, \mathrm{\Pi }$ and the advective part driven by the seepage velocity $\textbf{w}_{\textrm{FS}}$ of the fluid. For the diffusive part we apply a Fick approach for $\gamma ^\textrm{F}_{\textbf{w}_{\textrm{F}\beta \textrm{S}}}$ with a diffusivity parameter $\textrm{D}_{\textrm{fick}}$ for the external concentrations:

65 $$\begin{aligned} \begin{array}{lcll} \gamma ^\textrm{F}_{\textbf{w}_{\textrm{F}\beta \textrm{S}}}\,=\,-\displaystyle \frac{\textrm{R}\,\theta }{\mathrm{\textrm{D}}_{\textrm{fick}}\,\textrm{M}^{\textrm{F}\beta }_\textrm{mol}}. \end{array} \end{aligned}$$

In the next step we evaluate the advective part, applying the Cauchy stress of the fluid (61_(2)_) and the momentum production (62_(2)_). Analogous to the evaluation of the seepage velocity of the external components (64), we formulate the seepage velocity of the fluid by applying the conditions of the entropy inequality on the balance of momentum (14_(3)_):

66 $$\begin{aligned} \begin{array}{lcll} -\textrm{n}^{\textrm{F}}\,\textrm{grad}\lambda \,-\,\textrm{2}\,\gamma ^\textrm{F}_{\textbf{w}_{\textrm{FS}}}\,\textbf{w}_{\textrm{FS}}\,= & {} \textbf{0}. \end{array} \end{aligned}$$

The seepage velocity of the fluid follows with

67 $$\begin{aligned} \textrm{n}^{\textrm{F}}\textbf{w}_{\textrm{FS}}= \textbf{K}_\textrm{F} \, (-\textrm{grad}\,\lambda ). \end{aligned}$$

The constitutive relation for the advective part is based on the Darcy formulation, referring to a seepage velocity which is proportional to the hydraulic pressure $\lambda$. We apply the following ansatz, see Pierce et al. (2013b):

68 $$\begin{aligned} \begin{aligned} \textbf{K}_\textrm{F} =\textrm{k}_{\rm D}\,\displaystyle {(\frac{\textrm{n}^{\textrm{F}}}{1-\textrm{n}^{\textrm{S}}_{0S}})^{\textrm{m}_{\textrm{D}}}\,\textbf{M}^\mathbf{*}}, \hspace{1cm} \\ \text {with} ~ \textbf{M}^\mathbf{*} = \kappa \,\textbf{I} + \displaystyle {\frac{(\textrm{1}-3\kappa )}{\rm I_4}}\,\textbf{M}, \end{aligned} \end{aligned}$$

where $\textbf{M}^\mathbf{*}$ represents a positive definite material parameter tensor for the intrinsic hydraulic resistance of the cartilage solid and $\mathrm I_4$ a modified invariant. In liver tissue, the seepage velocity depends on the permeability of the solid structure and furthermore on the deformation of the solid, where $\textrm{k}_{\textrm{D}}$ describes the permeability and $\textrm{m}_{\textrm{D}}$ denotes a factor for the permeability deformation dependency. Inclusion of the volume fraction $\textrm{n}^{\textrm{F}}$ relates to the change of permeability caused by the change of pore space, where $\rm {n}^{\textrm{S}}_{0S}$ denotes the reference solid volume fraction. The development of fat vacuoles in the liver cell reduces the permeability, whereas areas with higher fluid conditions increase the motion of the fluid.
